# D-mannose alleviates intervertebral disc degeneration through glutamine metabolism

**DOI:** 10.1186/s40779-024-00529-4

**Published:** 2024-05-06

**Authors:** Zheng-Lin Dong, Xin Jiao, Zeng-Guang Wang, Kai Yuan, Yi-Qi Yang, Yao Wang, Yun-Tao Li, Tian-Chang Wang, Tian-You Kan, Jian Wang, Hai-Rong Tao

**Affiliations:** 1 Department of Orthopedics, Shanghai Key Laboratory of Orthopedic Implant, Shanghai Ninth People’s Hospital, Shanghai Jiao Tong University School of Medicine, Shanghai, 200011 China; 2 School of Medicine, Shanghai University, Shanghai, 200444 China

**Keywords:** D-mannose, Intervertebral disc degeneration, Thioredoxin-interacting protein (TXNIP), Glutamine

## Abstract

**Background:**

Intervertebral disc degeneration (IVDD) is a multifaceted condition characterized by heterogeneity, wherein the balance between catabolism and anabolism in the extracellular matrix of nucleus pulposus (NP) cells plays a central role. Presently, the available treatments primarily focus on relieving symptoms associated with IVDD without offering an effective cure targeting its underlying pathophysiological processes. D-mannose (referred to as mannose) has demonstrated anti-catabolic properties in various diseases. Nevertheless, its therapeutic potential in IVDD has yet to be explored.

**Methods:**

The study began with optimizing the mannose concentration for restoring NP cells. Transcriptomic analyses were employed to identify the mediators influenced by mannose, with the thioredoxin-interacting protein (*Txnip*) gene showing the most significant differences. Subsequently, small interfering RNA (siRNA) technology was used to demonstrate that *Txnip* is the key gene through which mannose exerts its effects. Techniques such as colocalization analysis, molecular docking, and overexpression assays further confirmed the direct regulatory relationship between mannose and TXNIP. To elucidate the mechanism of action of mannose, metabolomics techniques were employed to pinpoint glutamine as a core metabolite affected by mannose. Next, various methods, including integrated omics data and the Gene Expression Omnibus (GEO) database, were used to validate the one-way pathway through which TXNIP regulates glutamine. Finally, the therapeutic effect of mannose on IVDD was validated, elucidating the mechanistic role of TXNIP in glutamine metabolism in both intradiscal and orally treated rats.

**Results:**

In both in vivo and in vitro experiments, it was discovered that mannose has potent efficacy in alleviating IVDD by inhibiting catabolism. From a mechanistic standpoint, it was shown that mannose exerts its anti-catabolic effects by directly targeting the transcription factor max-like protein X-interacting protein (MondoA), resulting in the upregulation of TXNIP. This upregulation, in turn, inhibits glutamine metabolism, ultimately accomplishing its anti-catabolic effects by suppressing the mitogen-activated protein kinase (MAPK) pathway. More importantly, in vivo experiments have further demonstrated that compared with intradiscal injections, oral administration of mannose at safe concentrations can achieve effective therapeutic outcomes.

**Conclusions:**

In summary, through integrated multiomics analysis, including both in vivo and in vitro experiments, this study demonstrated that mannose primarily exerts its anti-catabolic effects on IVDD through the TXNIP-glutamine axis. These findings provide strong evidence supporting the potential of the use of mannose in clinical applications for alleviating IVDD. Compared to existing clinically invasive or pain-relieving therapies for IVDD, the oral administration of mannose has characteristics that are more advantageous for clinical IVDD treatment.

**Supplementary Information:**

The online version contains supplementary material available at 10.1186/s40779-024-00529-4.

## Background

Intervertebral disc degeneration (IVDD), a prevalent ailment affecting more than 500 million people worldwide with high relapse and disability rates, poses a substantial burden on public health and the global economy [1]. At present, within the clinical setting, conservative IVDD treatment strategies predominantly center on the use of medications aimed at alleviating pain and supporting nerve health. Advanced and deteriorating cases of IVDD may necessitate surgical intervention, but this approach carries the potential for substantial complications, including recurrence, infections, nerve damage, and adjacent segment disease [2]. It is essential to recognize that these treatment methods have considerable limitations in clinical practice and cannot directly address the underlying pathophysiological processes of IVDD. IVDD is characterized by an imbalance between anabolism and catabolism, which includes degradation of the extracellular matrix and compromised survival of nucleus pulposus (NP) cells [3, 4]. Among these, matrix metalloproteinases (MMPs) are the principal influencing factors responsible for extracellular matrix degradation in IVDD [5]. Hence, anti-MMP drugs have the potential to directly target the pathophysiological processes of IVDD, offering a tangible and effective therapeutic approach for treating IVDD [6]. Furthermore, the advancement of orally administered medications for delaying the degeneration of intervertebral discs will also improve patient adherence, rendering it a more convenient option for treating IVDD. Therefore, there is an urgent need for a biologically safe drug capable of targeting the disc lesion area and reversing the course of IVDD.

D-mannose (referred to as mannose) is a natural C-2 epimer of glucose. For an extended period, it was believed that the primary function of mannose was to glycosylate specific proteins, and individuals with congenital disorders of glycosylation type Ib were given mannose supplements to support their survival [7]. In recent investigations, mannose has emerged as a potent inhibitor of inflammation, particularly in the context of bone-related disorders. Moreover, it can alleviate osteoarthritis by inhibiting MMPs [8, 9]. Both IVDD and osteoarthritis are associated with similar extracellular matrix pathological processes, both of which are characterized by an imbalance in extracellular matrix metabolism, marked by excessive catabolic activation and anabolic suppression [10]. Nevertheless, it has yet to be determined whether mannose plays a role in IVDD and has therapeutic potential.

Thioredoxin-interacting protein (TXNIP), which can be induced by high glucose-isomer stimulation, was initially shown to be induced by 1,25-dihydroxyvitamin D_3_ in the leukemia cell line HL-60 [11, 12]. *Txnip* is a multifunctional gene involved in various cellular processes, such as the cell cycle, cell death, and metabolism [13]. Patients with decreased TXNIP levels due to *Txnip* gene mutations exhibit symptoms primarily characterized by lactic acidosis and low serum alanine levels [14]. However, the role of TXNIP in IVDD has not been thoroughly studied. Additionally, whether mannose can affect IVDD through TXNIP-mediated metabolism has not been systematically investigated.

In this study, our primary objective was to investigate the potential therapeutic effects of mannose on IVDD using primary rat NP cells. Additionally, we aimed to elucidate the underlying mechanisms through which mannose exerts its effects. Furthermore, we sought to evaluate the efficacy of the administration of mannose, both orally and via intradiscal injection, in alleviating IVDD in a rat model. Ultimately, through a combination of in vivo and in vitro experiments, we aimed to thoroughly validate the efficacy of mannose in treating IVDD and to provide a convenient oral treatment option for treating IVDD from a pathophysiological standpoint.

## Materials and methods

### Cell culture and treatment

To extract NP cells from the intervertebral discs of the rats, we euthanized 8-week-old rats (*n* = 42) (JieSiJie Laboratory Animal Company, Shanghai, China) via cervical dislocation. Subsequently, the rat tails were separated and disinfected with 75% (w/w) ethanol for 10 min. Afterward, the rat tail intervertebral discs were dissected using aseptic techniques with a sterile blade, and the NP tissue was extracted. The extracted tissue was then digested in 0.2% type II collagenase solution (Solarbio, Beijing, China) for 3 h. Following digestion, the separated cells were cultivated in full medium containing low-glucose DMEM. This medium was supplemented with 5% fetal bovine serum and 1% penicillin–streptomycin (Gibco, Thermo Fisher Scientific, MA, USA) and maintained at 37 °C in an environment with 5% CO_2_. This study was approved by the Ethical Review Approval of the Laboratory Animal Ethics Committee of the Shanghai Ninth People’s Hospital Affiliated to Shanghai Jiao Tong University School of Medicine for animal ethics experiments (SH9H-2023-A821-1).

### Cell Counting Kit-8 (CCK-8) cell proliferation assay

Initially, the cells were seeded onto 96-well plates at a precise density of 5 × 10^3^ cells/well to ensure uniform distribution and optimal growth conditions. This seeding density was meticulously chosen to strike a balance between ensuring sufficient cell coverage and preventing overcrowding. Following the seeding process, the cells were incubated for 24 h to acclimate to their new environment, facilitating adherence and initiating the early stages of growth. Subsequently, cellular proliferation was comprehensively assessed utilizing the CCK-8 assay (Sigma-Aldrich, MO, USA), a well-established and widely recognized method for evaluating cell viability and proliferation. To quantify cellular proliferation, the absorbance at 450 nm, which is indicative of metabolic activity and cell proliferation, was quantified using an Infinite M200 Pro multimode microplate reader (Tecan Group, Ltd., Männedorf, Switzerland). According to the concentration of mannose, this experiment was divided into groups with concentrations of 0, 10, 20, 40, and 80 mmol/L.

### 5-ethynyl-2’-deoxyuridine (EdU) staining

According to the instructions provided by the EdU assay kit (Ribobio, Guangzhou, China), firstly, the working solution of EdU contained in the kit was co-cultured with cells in a cell culture incubator. Subsequently, cells were fixed using polyoxymethylene and permeabilized with Triton X-100. Following the instructions, the reaction mixture contained in the kit was prepared and incubated with the cells. Finally, fluorescent microscopy (Leica Microsystems GmbH, Wetzlar, Germany) was used for microscopic observation. In this experiment, NP cells were respectively treated with mannose, IL-1β, and IL-1β + mannose.

### High-density culture and toluidine blue and alcian blue staining

To evaluate extracellular matrix levels, 3 × 10^5^ NP cells were suspended in 20 µl of complete medium (Gibco, Thermo Fisher Scientific, MA, USA) and seeded at the center of a 24-well plate. After 2 h of incubation at 37 °C, 0.5 ml of complete medium (Gibco, Thermo Fisher Scientific, MA, USA) was added. After an additional 24 h of incubation, the following treatments were performed. After 3 d, the cells were subjected to staining with toluidine blue solution (Solarbio, Beijing, China) and alcian blue solution (Solarbio, Beijing, China) for 1 h at room temperature. In this experiment, NP cells were respectively treated with IL-1β and IL-1β + mannose.

### RNA extraction and RT-qPCR

Total RNA was meticulously extracted from cultured cells using the AxyPrep Multisource RNA Miniprep Kit (Axygen, Corning, New York, USA). Briefly, after cell lysis, we performed centrifugation and washing steps using various reagents from the kit. Finally, we added TE buffer to dissolve the RNA, followed by centrifugation to obtain the RNA liquid sample. The synthesis of complementary DNA (cDNA) was conducted via reverse transcription using TaKaRa reverse transcription reagents (TaKaRa, Shiga, Japan). Subsequently, RT-qPCR assays were carried out utilizing the QuantStudio 6 Flex RT-qPCR System (Applied Biosystems, CA, USA) in conjunction with SYBR Green PCR Mix (Bimake, TX, USA). The detailed primer sequences are shown in Additional file 1: Table S1. In this experiment, NP cells were subjected to different treatments as required by the experimental demands, as detailed in the Results section.

### Western blotting analysis

For the extraction of total protein, cells were lysed in lysis buffer (Beyotime, Shanghai, China) containing a protein phosphatase inhibitor (Abmole Bioscience, TX, USA) for 15 min, followed by sonication. For the extraction of nuclear and cytoplasmic proteins, we utilized a Cell Nucleus and Cytoplasmic Protein Extraction Kit (Beyotime, Shanghai, China). In brief, cells were scraped using a cell scraper, and after centrifugation, the supernatant was removed. The cell cytoplasmic protein extraction reagent was added, followed by vigorous vortexing. Following centrifugation at 4 °C, the supernatant was collected to obtain the cytoplasmic protein fraction. For nuclear protein extraction, the cell nuclear protein extraction reagent was added to the centrifuged pellet mentioned above, followed by vigorous vortexing. The supernatant was harvested as the nuclear protein after centrifugation at 4 °C. The obtained protein solution was combined with loading buffer (Beyotime, Shanghai, China) and subjected to incubation at 99 °C for 10 min. Subsequently, proteins were separated using 4‒20% ExpressPlus™ PAGE Gel (GenScript, Nanjing, China) and electrophoresed in Tris-MOPS-SDS Running Buffer (GenScript, Nanjing, China) diluted with ddH_2_O. Following gel electrophoresis, the protein bands were transferred onto 0.22-µm PVDF membranes (Millipore Sigma, MA, USA) through electroblotting. The membranes were blocked with 5% BSA-TBST [tris-buffered saline (TBS)-0.1% Tween 20] (Beyotime, Shanghai, China) at room temperature for 1 h. After three TBST washes, as described for our research, primary antibodies against MMP1, MMP3, MMP9, MMP13, collagen II, MYC, mannose phosphate isomerase (MPI), TXNIP, glutamate dehydrogenase 1 (GLUD1, also known as GDH), p38, p-p38, c-jun N-terminal kinase (JNK), p-JNK, extracellular signal-regulated kinases (ERK), p-ERK, β-tubulin, Histone H3, and β-actin (Proteintech, Wuhan, China); max-like protein X-interacting protein (MLXIP, also known as MondoA) (ABclonal, Wuhan, China); and solute carrier family 1 member 5 (SLC1A5; CST, MA, USA) were added at 4 °C overnight. Subsequently, the membranes were incubated with the corresponding species-specific HRP-conjugated anti-rabbit/mouse secondary antibodies (Proteintech, Wuhan, China). After treatment with ultrasensitive enhanced chemiluminescence detection solution (NCM, Suzhou, China), protein immunoreactivity was detected using an eBlot Touch Imager system (e-Blot, Shanghai, China). In this experiment, NP cells were subjected to different treatments as required by the experimental demands, as detailed in the Results section.

### Cell immunofluorescence

The cells were immobilized by treatment with prechilled 4% paraformaldehyde for 15 min and then incubated with 0.5% Triton for 5 min. After blocking with an immunofluorescence blocking solution (Beyotime, Shanghai, China) for 1 h, the cells were incubated with primary antibodies against MMP3, MMP13, and MondoA (Proteintech, Wuhan, China) overnight at 4 °C. The next day, the samples were washed three times with TBST. Subsequently, the cells were treated with a fluorescein isothiocyanate (FITC)/Cy3-conjugated anti-rabbit secondary antibody (Proteintech, Wuhan, China) at room temperature for 1 h. Then, the nuclei were stained with 4’,6-diamidino-2-phenylindole (DAPI) working solution (Beyotime, Shanghai, China) for 10 min, and the cellular cytoskeleton was stained with CoraLite^®^ Plus 488-phalloidin (Proteintech, Wuhan, China) for 20 min. Images were captured using either a Leica confocal scanning microscope or a DM4000 B epifluorescence microscope (Leica Microsystems GmbH, Wetzlar, Germany). In this experiment, NP cells were respectively treated with IL-1β, and IL-1β + mannose.

### Transcriptomics and data processing

Transcriptomic analysis was performed by Wuhan MetWare Biotechnology Co., Ltd. (www.metware.cn). Total RNA was extracted with TRIzol (Ambion, Thermo Fisher Scientific, MA, USA) and normalized to 3 μg for library preparation. Sequencing libraries were generated using the NEBNext^®^ Ultra™ RNA Library Prep Kit for Illumina^®^ (NEB, USA) in strict accordance with the manufacturer’s protocols. Index codes were incorporated to distinguish between samples during library preparation. Library fragments, ideally ranging from 250 to 300 bp, were purified utilizing the AMPure XP system (Beckman Coulter, Beverly, USA) and subjected to quality assessment on a Bioanalyzer 210. Illumina sequencing was used to analyze the generated double-stranded cDNA libraries. The RNA sequence data were comprehensively analyzed using HISAT2 v2.0, followed by read assembly utilizing StringTie (v1.3.3b) through a reference-based approach. Gene expression levels were quantified using Feature Counts v1.5.0-p3. Statistical significance was determined by adjusting the *P-*values of genes using the Benjamini and Hochberg method. Genes meeting the criteria of a corrected *P* ≤ 0.05 and a |log_2_ fold change (FC)|≥ 0.5 were classified as differentially expressed genes (DEGs). Furthermore, enrichment analysis of Kyoto Encyclopedia of Genes and Genomes (KEGG) pathways and Gene Ontology (GO) was conducted employing advanced algorithms within the R package. In this experiment, NP cells were respectively treated with IL-1β and IL-1β + mannose.

### siRNA and plasmid transfection

si-NC, si-Txnip and si-Myc were obtained from Generalbiol Company (Anhui, China). Prior to transfection, the cells were seeded in a 6-well plate and allowed to reach 60‒80% confluency. Subsequently, Lipofectamine™ RNAiMAX Transfection Reagent (Thermo Fisher Scientific, MA, USA) was used for transfection according to the manufacturer’s instructions. In brief, siRNAs at a concentration of 10 μmol/L and RNAiMAX reagent were separately diluted in Opti-MEM (Gibco, Thermo Fisher Scientific, MA, USA). The two solutions were then mixed in equal proportions at room temperature for 5 min and added to a 6-well plate. The gene knockdown effects were verified by Western blotting. All experiments involving si-Txnip and si-Myc were conducted with si-NC included as a control in the non-si-Txnip and non-si-Myc groups.

oe-NC and oe-MPI [based on pcDNA3.1(+)] were obtained from Generalbiol Company (Anhui, China). Prior to transfection, the cells were ensured to reach 70‒90% confluency within a 6-well plate. Lipofectamine™ 3000 Transfection Reagent (Thermo Fisher Scientific, MA, USA) was subsequently used for transfection according to the manufacturer’s instructions. Briefly, plasmids were diluted to a concentration of 5 μg/μl, along with P3000™ Reagent and Lipofectamine™ 3000 Reagent, in Opti-MEM (Gibco, Thermo Fisher Scientific, MA, USA) according to the manufacturer’s guidelines. These solutions were then mixed at room temperature for 10‒15 min before being added to a 6-well plate. The efficacy of gene overexpression was confirmed through Western blotting experiments. All overexpression trials included oe-NC as a control within the non-oe-MPI groups. The detailed primer sequences are shown in Additional file 1: Table S2.

In this experiment, NP cells were subjected to different treatments as required by the experimental demands, as detailed in the Results section.

### Single‐cell RNA statistical analysis

For this investigation, single-cell RNA sequencing (scRNA-seq) data from the GSE165722 dataset from the Gene Expression Omnibus (GEO) database were retrieved and analyzed with the Seurat package (v4.3.0) in R. After the data were downloaded, the data were normalized, highly variable genes were identified, and scaling procedures were performed. Principal component analysis (PCA) was then performed on the scaled data, with the determination of the number of principal components accomplished through elbow plot analysis. To account for batch effects between the degeneration grade groups (Grade II and III as group 1 and Grade IV and V as group 2), Harmony, an algorithm for the integration of multiple datasets, was employed. The PCA embeddings were harmonized by incorporating the “group” variable and visualized using a dimensionality reduction plot and violin plot. Subsequently, the data were further analyzed by conducting uniform manifold approximation and projection (UMAP) and t-distributed stochastic neighbor embedding (t-SNE) on the harmonized embeddings. This step was followed by nearest neighbor identification and cluster detection. Marker genes for all clusters were identified, and the results were exported for further analysis. The cell type and degeneration grade group information were combined for each cell to facilitate intergroup differential expression analysis. Finally, the expression of the gene of interest, *Txnip*, was visualized within each cell using violin and feature plots, providing a clear representation of the gene expression pattern across different degeneration grades. These plots were generated by splitting the data by the “group” variable.

### Molecular docking

The experimental workflow commenced with the adjustment of the protonation state of the compounds to pH = 7.4, ensuring physiological relevance. Subsequently, the three-dimensional structures of the compounds were generated utilizing Open Babel, a powerful computational tool for chemical structure manipulation. AutoDock Tools (ADT3) were then employed to meticulously prepare and parameterize both the receptor protein and ligands, ensuring accurate representation for subsequent docking simulations. Docking grid files were meticulously crafted through AutoGrid of sitemap, a process vital for defining the spatial constraints of the binding site. The subsequent docking simulations were executed using AutoDock Vina (1.2.0), a state-of-the-art molecular docking software renowned for its accuracy and efficiency in predicting ligand-receptor interactions. Following the docking simulations, the most favorable pose of each ligand within the binding site was meticulously selected for subsequent interaction analysis. PyMOL, a versatile molecular visualization tool, was then harnessed to construct the protein–ligand interaction diagram. In this comprehensive representation, the MondoA protein was depicted as a slate-colored cartoon model, providing a structural context for understanding ligand binding. Moreover, the ligands are represented in cyan, clearly delineating their molecular structure and orientation within the binding site. The critical binding sites are highlighted in magenta, facilitating visualization of key interaction points. To streamline the representation, nonpolar hydrogen atoms were omitted, with a focus on the essential polar and nonpolar interactions driving ligand binding. Hydrogen bonds, ionic interactions, and hydrophobic interactions are illustrated as yellow, magenta, and green dashed lines, respectively, providing comprehensive insights into the molecular mechanisms underlying ligand-receptor interactions.

### “TM” widely targeted metabolomics and data processing

“TM” widely targeted metabolomics, provided by Wuhan MetWare Biotechnology Co., Ltd. (www.metware.cn), was used in this study. After initiating the experimental process, cell samples were promptly isolated from the culture medium and then thawed on ice. Following a repeated vortexing cycle, intracellular metabolites were extracted by adding 300 μl of cold ACN/H_2_O (75:25, v/v) three times. After centrifugation for 15 min, the supernatants were carefully transferred to fresh 1.2 ml polypropylene tubes and promptly preserved at -80 °C for 15 min. Subsequently, the samples underwent a 3 h freeze-drying process. The resulting residues were dissolved in 80 μl of ACN/H_2_O (20:80, v/v) and centrifuged for 15 min (13,000 rpm, 4 °C). A 60 μl aliquot of supernatant from each sample was then prepared for subsequent ultrahigh-performance liquid chromatography-mass spectrometry (UHPLC-MS) analysis, with random injections. Metabolomic analysis was carried out with an UltiMate 3000 UHPLC system coupled with a Q Exactive™ Hybrid Quadrupole-Orbitrap mass spectrometer for high-resolution mass spectrometry. The metabolites were separated through an ACQUITY UPLC HSS T3 column at 40 °C using a mobile phase comprising solvent A (0.1% formic acid in water) and solvent B (acetonitrile) at a flow rate of 0.3 ml/min. The elution gradient was as follows: 0–1 min: 2% B, 1–19 min: 2–100% B, 19–21 min: 100% B, and 21–25 min: 2% B. Each sample was injected with a 5 μl volume, and mass spectrometry was conducted in both ESI(+) and ESI(−) modes. The data analysis procedure began with the transformation of the raw UPLC-MS data into the mzXML format. Following this, the data underwent essential preprocessing steps, including correction for nonlinear retention time, filtration of peaks, and extraction of relevant information. These tasks were executed using the XCMS package within the R environment. Subsequently, the resulting dataset, comprising crucial parameters such as the mass-to-charge ratio (m/z), retention time, and ion intensity, underwent additional refinement and comprehensive analysis through the metaX package in R. Signal correction and peak normalization were executed based on quality control (QC) samples, with metabolites displaying a coefficient of variation (CV) exceeding 30% in the QC samples excluded. Batch normalization of the peak area was then performed to facilitate cross-sample comparison. Multivariate statistical analysis was carried out using SIMCA-P 14.1 software, employing PCA to discriminate between the control and drug-treated groups. Differentially abundant metabolites were identified based on a variable importance in projection (VIP) ≥ 1 and a *P* ≤ 0.05, with consultation of databases such as METLIN and HMDB. Some metabolites were further validated by matching their MS/MS spectra and retention times with those of commercially available standards. For visualization purposes, the heatmap package in R was utilized to cluster differentially abundant metabolites among all groups. Additionally, relevant metabolic pathways were elucidated through pathway enrichment analysis using MetaboAnalyst 4.0 to reveal significant alterations induced by shikonin. In this experiment, NP cells were respectively treated with IL-1β and IL-1β + mannose.

### Integrated analysis of the metabolomic and transcriptomic data

Integration of the metabolomics and transcriptomics datasets was achieved via MetScape software, aiming to explore potential associations between DEGs and significantly altered metabolites.

### Single-gene-based bulk correlation and gene set enrichment analysis (GSEA)

In this study, a single-gene-based bulk correlation analysis was employed to generate a gene list for GSEA. Initially, expression data for all the measured genes were acquired. The stats package in R (v4.2.2) was used to calculate Pearson correlation coefficients between each gene and a specific gene of interest. The correlation coefficients were then considered log_2_ FCs, with positive and negative values indicating direct and inverse correlations, respectively. This method allowed for the generation of a gene list, ranked based on correlation coefficients, thus accounting for the variation in gene expression in relation to the gene of interest. Subsequently, GSEA was carried out using the gseGO function from the clusterProfiler package (v4.6.2). Adjustments for multiple comparisons of *P-*values were made using the Benjamini–Hochberg method. Predefined MYC target V1 and V2 gene sets were used for GSEA. These gene sets were imported from existing files and did not necessitate any additional manipulation. The enrichment process was visually represented through an enrichment plot generated using the ggplot2 package (v3.4.0). The plot displays the significantly enriched gene sets and allows for comprehensive visualization of the enrichment process.

### Measurement of intracellular adenosine triphosphate (ATP) levels

An ATP assay kit (Beyotime, Shanghai, China) was used to assess intracellular ATP levels. The cells were lysed and subsequently centrifuged at 4 °C and 12,000 × *g*, after which the supernatant was collected. A gradient dilution of ATP standard solution was prepared. Subsequently, the prepared ATP detection reagent was added sequentially to the samples and the diluted ATP standard solution. A GloMax^®^ 20/20 luminometer (Promega, WI, USA) was used to measure luminescence units. The intracellular ATP level was determined based on the final standard curve prepared from the gradient-diluted ATP standard solution. In this experiment, NP cells were respectively treated with glutamine, IL-1β, IL-1β + glutamine, IL-1β + mannose, or IL-1β + mannose + glutamine.

### Measurement of intracellular glutamine and glutamate levels

We used an intracellular glutamine assay kit (Dojindo, Tokyo, Japan) and a glutamate assay kit (Solarbio, Beijing, China) to determine the concentrations of the respective substances within the cells. In brief, after cell lysis, centrifugation was performed to collect the supernatant. Subsequently, gradient dilution was carried out using standard solutions. Then, the absorbance of the gradient-diluted standard solutions and samples was measured using an Infinite M200 Pro multimode microplate reader (Tecan Group, Ltd., Männedorf, Switzerland). A standard curve was constructed based on the absorbance values of the gradient-diluted standard solutions. The level of the corresponding substance in the samples was predicted using the standard curve. In this experiment, NP cells were respectively treated with si-NC, IL-1β + si-NC, IL-1β + mannose + si-NC, IL-1β + mannose + si-Txnip, or IL-1β + mannose + si-Txnip + si-Myc.

### Animal models

This study was approved by the Ethical Review Approval of the Laboratory Animal Ethics Committee of the Shanghai Ninth People’s Hospital Affiliated to Shanghai Jiao Tong University School of Medicine for animal ethics experiments (SH9H-2023-A821-1). We ordered 18 rats for an 11-week period, followed by a 1-week acclimation period during which the rats were allowed to adapt to their environment. Briefly, the rats were anesthetized, and a 21-gauge needle was inserted 3.0 mm into the intervertebral disc of the rat tail for 30 s to establish the IVDD model [15].

To minimize the number of rats used, the mannose intervertebral disc injection treatment group underwent a single puncture through three intervertebral disc segments in sequence (C_6_/C_7_–C_8_/C_9_) [16]. Six rats were allocated to the intervertebral disc injection group. These sections were designated the puncture section, puncture + mannose injection section, and puncture + mannose + glutamine injection section. To further directly verify the effects of mannose and glutamine on IVDD, after 2 weeks of fine needle puncture (FNP) treatment, we administered intradiscal injections of PBS (5 μl), 40 mmol/L mannose (5 μl), and a mixture of 40 mmol/L mannose and 8 mmol/L glutamine solution (5 μl) every week using 31-gauge needles.

In the gavage group, only a single intervertebral disc segment (C_6_/C_7_) of the tail vertebra was punctured into each rat. Twelve rats were assigned to the gavage group, with 6 rats allocated to treatment group and 6 rats to control group. After modeling, the mice in the treatment group were administered 80 mmol/L mannose solution daily, with a dose of 2 g/kg of mannose delivered through oral gavage every 2 d. The control group received regular drinking water daily, and an equivalent volume of drinking water via oral gavage was given to the treatment group every 2 d.

Six or eight weeks after the operation, the rats were euthanized under anesthesia. Then, their tails were dissected and prepared for imaging and histological evaluation.

#### Radiographic analysis

We performed digital X-ray imaging of the rat tail vertebrae in accordance with the manufacturer’s instructions utilizing a 21 lp/mm detector that allowed for a maximum geometric magnification of 5X (Faxitron VersaVision; Faxitron Bioptics LLC, AZ, USA). In this experiment, the experimental groups consisted of two types: one included the sham group, FNP group, FNP + mannose group, and FNP + mannose + glutamine group; the other included the sham + H_2_O group, FNP + H_2_O group, sham + mannose group, and FNP + mannose group.

#### Histological staining, safranin O (SO) staining, immunohistochemistry (IHC) and tissue immunofluorescence staining

Mouse tail tissue was fixed in 4% paraformaldehyde for more than 7 d, dehydrated, embedded in wax, and sectioned at a thickness of 4 μm. Next, the paraffin-embedded sections were subjected to deparaffinization. Subsequently, the sections were subjected to hematoxylin and eosin (HE) staining using Harris hematoxylin and eosin dye (Ribiology, Shanghai, China), as well as SO staining using fast red and alcian green dye solutions (Ribiology, Shanghai, China). For IHC, the sections were initially subjected to antigen retrieval using citrate buffer (Ribiology, Shanghai, China), followed by treatment with a hydrogen peroxide solution. After blocking with BSA, the sections were sequentially incubated with primary antibodies against MMP13 (Proteintech, Wuhan, China), collagen II (Arigo, MA, USA), and SLC1A5 (Affinity, OH, USA) and secondary antibodies (Abcam, Cambridge, UK). Finally, DAB chromogenic solution (DAKO, CA, USA) was applied for visualization. The cell nuclei were counterstained with Harris hematoxylin (Ribiology, Shanghai, China). For tissue immunofluorescence staining, the sections were subjected to antigen retrieval with EDTA (Ribiology, Shanghai, China), followed by blocking with BSA. After incubation with primary antibodies against TXNIP and MondoA (Proteintech, Wuhan, China) and secondary antibodies (Abcam, Cambridge, UK), the nuclei were counterstained with DAPI before microscopic examination and imaging. In this experiment, the experimental groups consisted of two types: one included sham group, FNP group, FNP + mannose group, and FNP + mannose + glutamine group; the other included sham + H_2_O group, FNP + H_2_O group, sham + mannose group, and FNP + mannose group.

### Statistical analysis

The data are displayed as the mean ± standard deviation (SD), and the results are visualized using bar graphs with individual data points. Color intensity measurements of the images were performed using ImageJ v1.8.0 software (National Institutes of Health). Statistical analyses were carried out using Prism Version 9 Software (GraphPad, CA, USA) and SPSS 22.0 (IBM, New York, USA). Unpaired two-tailed Student’s *t*-tests were used for pairwise comparisons between two groups. For comparisons among three or more groups, one-way analysis of variance (ANOVA) was used, followed by Tukey’s post hoc test. Nonparametric tests involving comparisons among multiple groups of ordered categorical variables were utilized for analyses. In the main text, only the statistical results relevant to the demonstration are highlighted. *P* < 0.05 was considered to indicate a statistically significant difference.

## Results

### Mannose inhibits catabolism in interleukin-1β (IL-1β)-treated rat NP cells

**Fig. 1:**
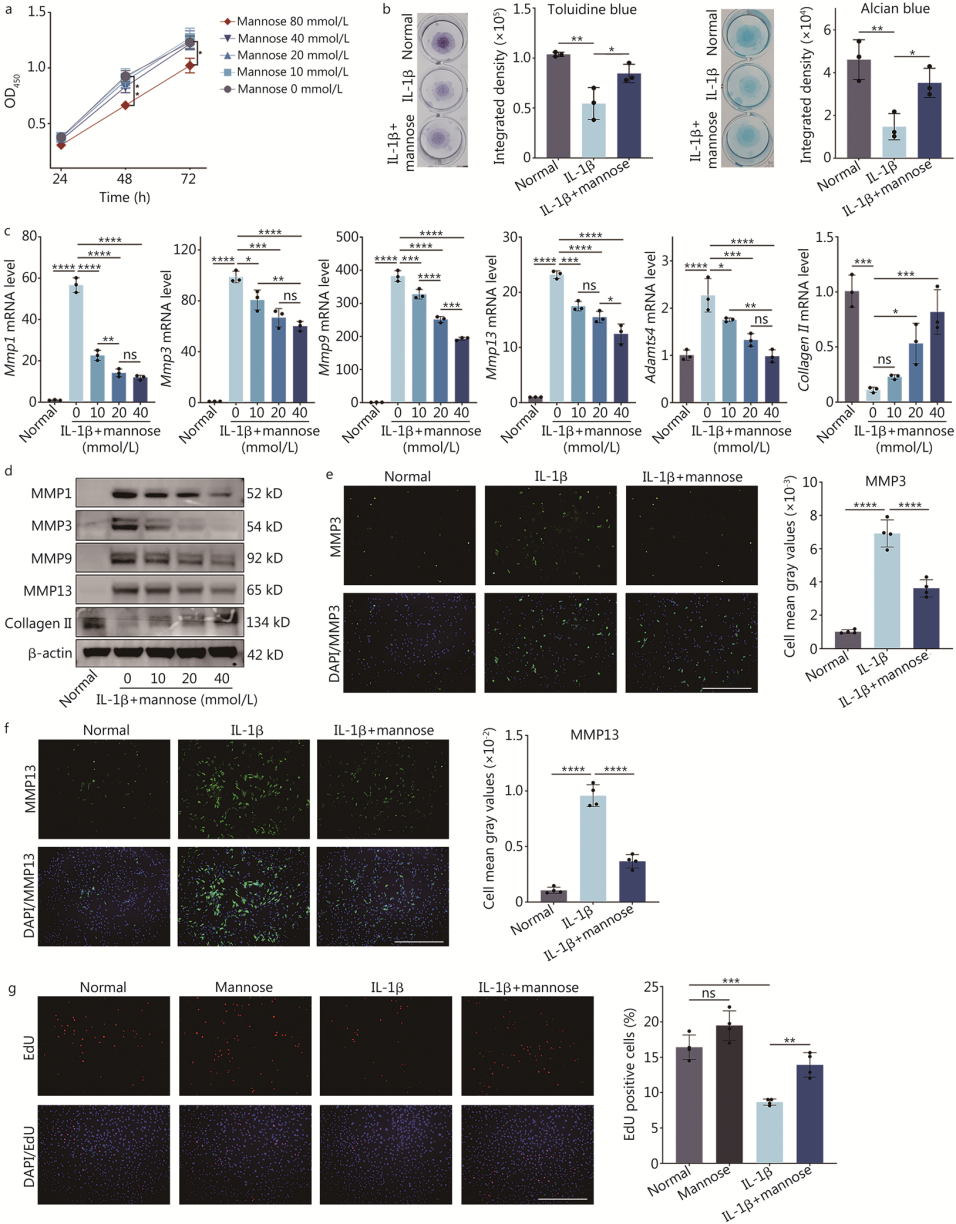
Mannose suppresses catabolism in rat nucleus pulposus (NP) cells treated with IL-1β. **a** Effect of different concentrations of mannose on cell viability at various time points, as determined by a CCK-8 assay. The statistically significant differences between the 80 mmol/L and 0 mmol/L groups are shown. ^*^P < 0.05. **b** Toluidine blue and alcian blue staining of NP cells treated with IL-1β and IL-1β + mannose. **c** RT-qPCR analysis of *Mmp1*, *Mmp3*, *Mmp9*, *Mmp13*, *Adamts4* and *collagen II* in NP cells treated with different concentrations of mannose. **d** Western blotting of MMP1, MMP3, MMP9, MMP13 and collagen II in NP cells treated with different concentrations of mannose.** e** Immunofluorescence and quantitative analysis of MMP3 in NP cells treated with IL-1β and IL-1β + mannose (original magnification × 100; scale bar = 600 µm; *n* = 4). **f** Immunofluorescence and quantitative analysis of MMP13 in NP cells treated with IL-1β and IL-1β + mannose (original magnification × 100; scale bar = 600 µm; *n* = 4). **g** EdU staining of NP cells treated with mannose, IL-1β, and IL-1β + mannose (original magnification × 100; scale bar = 600 µm; *n* = 4). Unless otherwise specified, the IL-1β concentration was 10 ng/ml, the mannose concentration was 40 mmol/L, and *n* = 3. ^*^P < 0.05, ^**^P < 0.01, ^***^P < 0.001, ^****^P < 0.0001. ns non-significant, CCK-8 cell counting kit-8, IL-1β interleukin-1β, MMP1 matrix metalloproteinase 1, MMP3 matrix metalloproteinase 3, MMP9 matrix metalloproteinase 9, MMP13 matrix metalloproteinase 13, ADAMTS4 a disintegrin and metalloproteinase with thrombospondin motifs 4, EdU 5-ethynyl-2’-deoxyuridine, DAPI 4’,6-diamidino-2-phenylindole

First, a CCK-8 assay was performed to evaluate the effect of 0, 10, 20, 40, and 80 mmol/L mannose on the viability of primary NP cells within a 72-h timeframe, revealing that the maximum concentration for NP cells without toxicity was 40 mmol/L (Fig. 1a). Subsequent experiments were conducted using a concentration of 40 mmol/L for 24 h, unless stated otherwise. After treating NP cells with IL-1β and IL-1β + mannose for 3 d, the extracellular matrix was evaluated using toluidine blue and alcian blue staining. Mannose significantly alleviated the loss of the extracellular matrix caused by IL-1β (Fig. 1b). The RT-qPCR results revealed dose-dependent and extensive impacts of mannose on *Mmp1*, *3*, *9*, *13*, and *Adamts4*. Additionally, RT-qPCR results demonstrated that at concentrations exceeding 20 mmol/L, mannose reversed the loss of *collagen II* (Fig. 1c). In line with the findings from the RT-qPCR results, the Western blotting results also revealed dose-dependent and extensive effects of mannose on MMP1, 3, 9, 13 (Fig. 1d; Additional file 1: Fig. S1). Moreover, at concentrations greater than 20 mmol/L, mannose reversed the loss of collagen II (Fig. 1d; Additional file 1: Fig. S1). To further validate the anti-MMP effects of mannose, immunofluorescence was used to assess the expression of MMP3 and MMP13 in the NP cells. Mannose still demonstrated remarkable efficacy in suppressing MMPs (Fig. 1e, f). Finally, a EdU staining experiment was conducted to investigate the impact of mannose on cell proliferation. The results indicated that mannose significantly increased the proliferation of NP cells treated with IL-1β. However, while mannose also increased the proliferation of normal NP cells, the difference was not statistically significant (Fig. 1g).

In conclusion, the in vitro experiments demonstrated that mannose significantly suppressed matrix degradation metabolism, promoted matrix synthesis metabolism, and enhanced cell proliferation in a concentration-dependent manner.

### Mannose suppresses catabolism through TXNIP

**Fig. 2:**
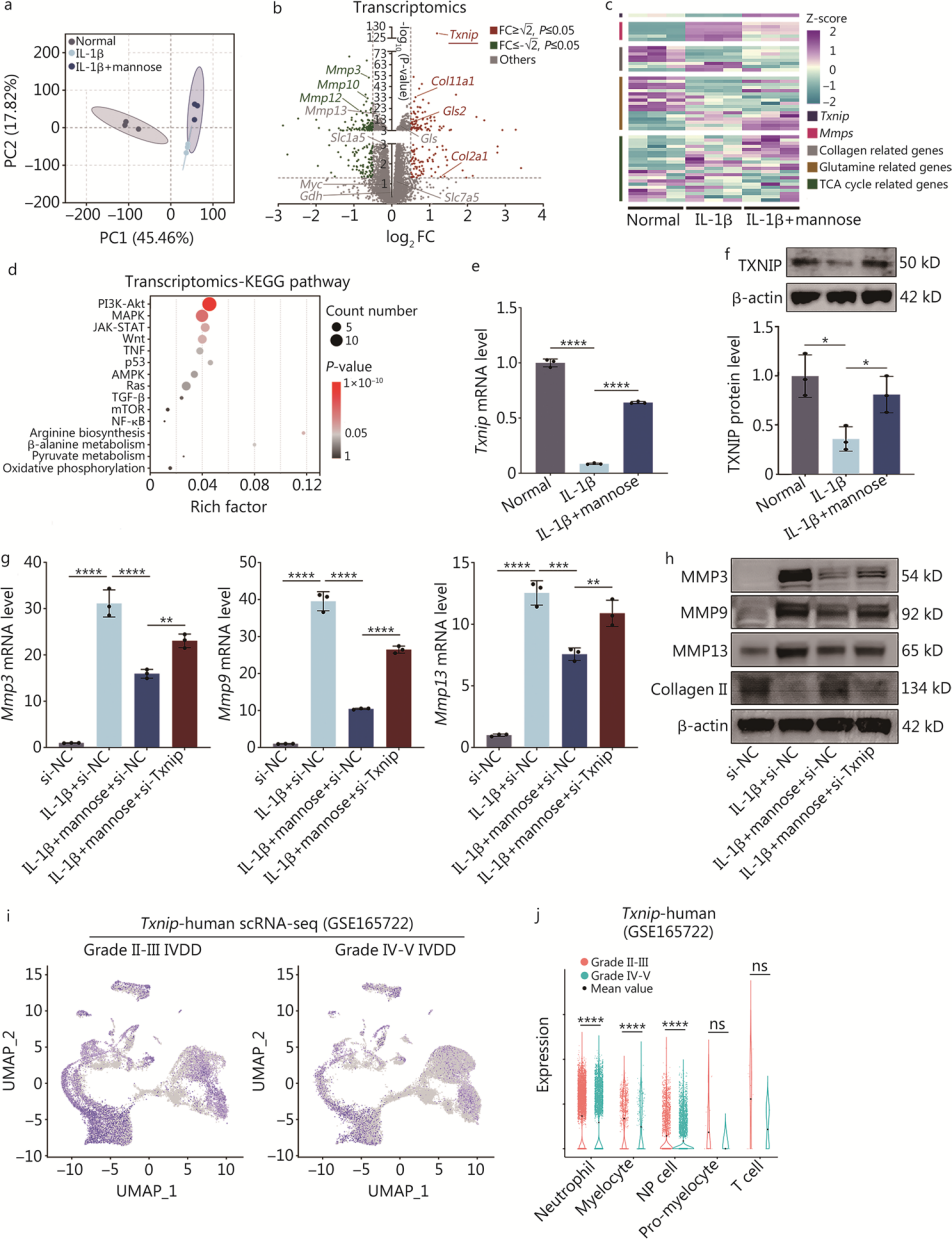
Mannose inhibits catabolism by regulating TXNIP. **a** Principal component analysis (PCA) of normal group, IL-1β group and IL-1β + mannose group. **b** Transcriptome volcano plot of IL-1β group and IL-1β + mannose group. **c** Gene heatmap comparing IL-1β group and IL-1β + mannose group. **d** Transcriptome KEGG pathway ranking between IL-1β group and IL-1β + mannose group. RT-qPCR (**e**) and Western blotting (**f**) analysis of TXNIP in NP cells treated with IL-1β and IL-1β + mannose. RT-qPCR (**g**) and Western blotting (**h**) analysis of MMP3, MMP9, MMP13 and collagen II in NP cells treated with si-NC, IL-1β + si-NC, IL-1β + mannose + si-NC or IL-1β + mannose + si-Txnip. **i** UMAP plots of human single-cell RNA sequencing (scRNA-seq) data (GSE165722) showing differences in *Txnip* expression between Grade II–III and Grade IV–V intervertebral disc degeneration (IVDD) patients (*n* = 4). **j** The expression of *Txnip* in different cell types according to human scRNA-seq (GSE165722) between Grade II–III and Grade IV–V IVDD patients. Unless otherwise specified, 10 ng/ml IL-1β and 40 mmol/L mannose were used, *n* = 3. ^*^P < 0.05, ^**^P < 0.01, ^***^P < 0.001, ^****^P < 0.0001. ns non-significant, IL-1β interleukin-1β, MMP3 matrix metalloproteinase 3, MMP10 matrix metalloproteinase 10, MMP12 matrix metalloproteinase 12, MMP13 matrix metalloproteinase 13, SLC1A5 solute carrier family 1 member 5, GDH glutamate dehydrogenase 1, SLC7A5 solute carrier family 7 member 5, Col2a1 collagen type II alpha 1 chain, GLS glutaminase, Gls2 glutaminase 2, Col11a1 collagen type XI alpha 1 chain, TXNIP thioredoxin-interacting protein, KEGG Kyoto Encyclopedia of Genes and Genomes, PI3K-Akt phosphatidylinositol-3 kinase-protein kinase B, MAPK mitogen-activated protein kinase, JAK-STAT janus kinase-signal transducer and activator of transcription, WNT wingless/integrated, TNF tumor necrosis factor, AMPK AMP-activated protein kinase, Ras rat sarcoma protein, TGF-β transforming growth factor β, mTOR mammalian target of rapamycin, NF-κB nuclear factor kappa-light-chain-enhancer of activated B cells, MMPs matrix metalloproteinases, TCA tricarboxylic acid cycle

The PCA results revealed distinct differences in the transcriptomes among the three groups (Fig. 2a). In summary, there were 1804 DEGs [|log_2_ FC|≥ 0.5 and false discovery rate (FDR) ≤ 0.05] between normal group and IL-1β group. Additionally, 401 DEGs were differentially expressed between IL-1β group and IL-1β + mannose group. Among them, 192 DEGs exhibited significant changes in both aforementioned comparisons (Additional file 1: Fig. S2a). Through volcano plots, heatmaps, and GO analysis, differential gene expression was further explored, revealing that mannose exhibited anti-MMP effects and was effective at rescuing the extracellular matrix (Fig. 2b, c; Additional file 1: Fig. S2b). Moreover, notable changes were observed in KEGG pathways linked to MMPs, such as the phosphoinositide 3-kinase (PI3K), mitogen-activated protein kinase (MAPK), and janus kinase-signal transducer and activator of transcription (JAK-STAT) pathways (Fig. 2d) [17]. Furthermore, in the comparison between IL-1β group and IL-1β + mannose group, *Txnip* exhibited the lowest *P-*value [-log_10_ (*P-*value) = 127.1816] compared to the other genes (Fig. 2b). Subsequently, RT-qPCR and Western blotting experiments confirmed that the expression of TXNIP decreased under IL-1β stimulation and mannose significantly restored the expression of TXNIP in vitro (Fig. 2e, f). To demonstrate the role of TXNIP in MMPs, a small interfering RNA targeting *Txnip* (si-Txnip) was utilized to knockdown *Txnip* in NP cells (Additional file 1: Fig. S3a). After *Txnip* knockdown, the excellent anti-MMP effects of mannose and its ability to rescue collagen II were significantly inhibited (Fig. 2g, h; Additional file 1: Fig. S3b). To verify whether the expression of TXNIP in human IVDD corresponds to the expression observed in rat NP cells stimulated with IL-1β, single-cell sequencing analysis of intervertebral disc tissues from patients with Grade II to V IVDD was conducted using the GEO dataset (GSE165722). The results revealed a decrease in *Txnip* expression in all cell groups and a significant decrease in *Txnip* expression in neutrophil, myelocyte and NP cell groups. Thus, there was a correlation between *Txnip* downregulation and the progression of IVDD (Fig. 2i, j). In addition, to examine whether the decrease in *Txnip* expression under conditions of prolonged inflammatory disease exhibits a general trend, published data and the GEO database were utilized to determine the expression of *Txnip* in chondrocytes and synovial tissues from humans, rats and mice with osteoarthritis (Additional file 1: Fig. S4), which has a similar pathological and physiological process to IVDD [18]. The results consistently showed a significant decrease in TXNIP expression over the course of prolonged inflammation.

In summary, the findings revealed that mannose exerts its anti-catabolic effects by upregulating TXNIP. It was also discovered that under conditions of chronic inflammation, at least in diseases such as IVDD and osteoarthritis, the expression of TXNIP decreases.

### Mannose activates TXNIP by directly targeting the MondoA transcription factor

**Fig. 3:**
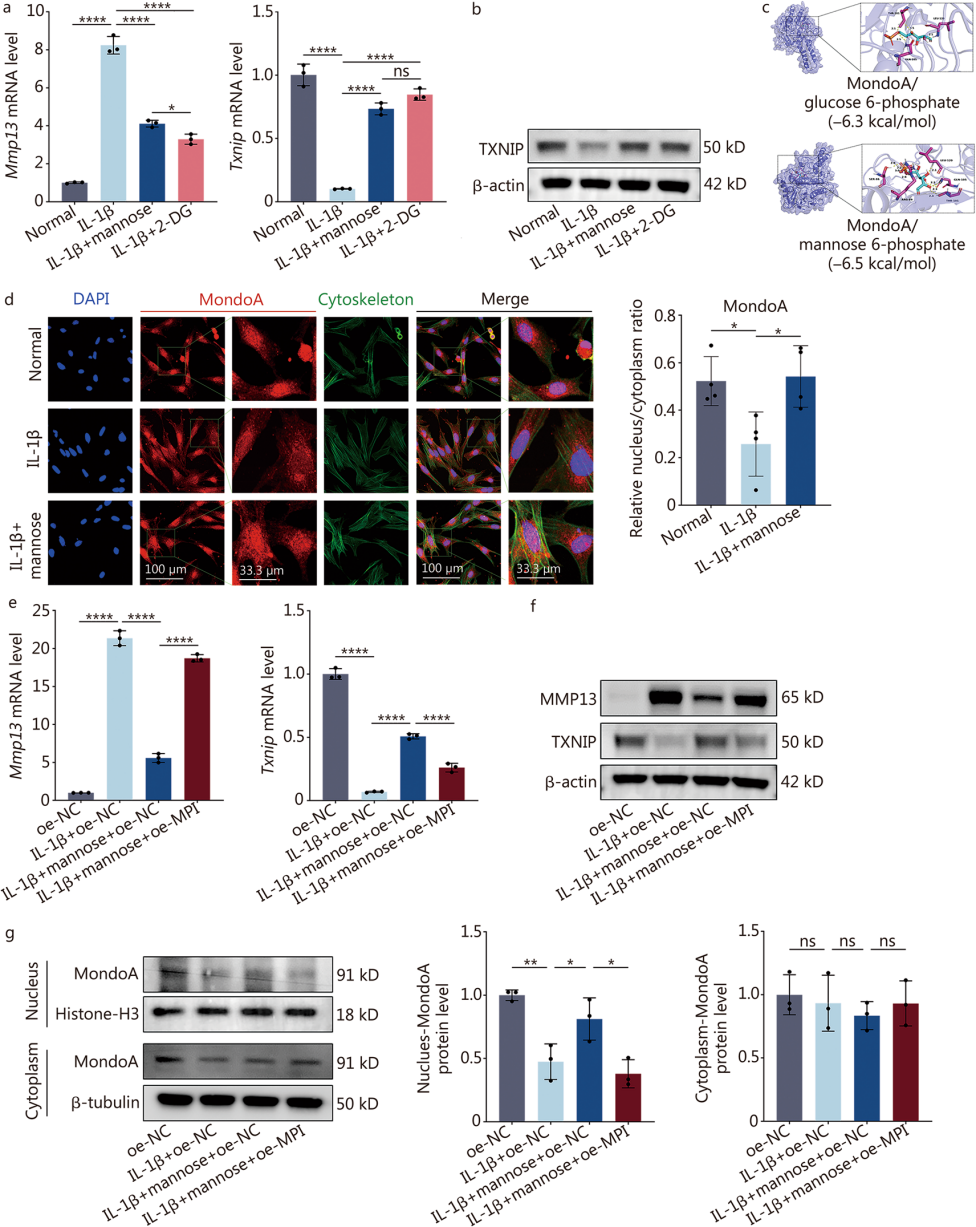
Mannose regulates TXNIP by directly targeting the MondoA transcription factor. **a** RT-qPCR analysis of *Mmp13* and *Txnip* in NP cells treated with IL-1β, IL-1β + mannose or IL-1β + 2-DG (2 mmol/L). **b** Western blotting analysis of TXNIP in NP cells treated with IL-1β, IL-1β + mannose or IL-1β + 2-DG (2 mmol/L). **c** MondoA molecular docking experiments with glucose 6-phosphate and mannose 6-phosphate. **d** Confocal microscopy and quantitative analysis of the intracellular distribution of MondoA in NP cells treated with IL-1β and IL-1β + mannose (original magnification × 400, × 3600; scale bar = 100 µm, 33.33 µm; *n* = 4). RT-qPCR (**e**) and Western blotting (**f**) analysis of MMP13 and TXNIP in NP cells treated with oe-NC, IL-1β + oe-NC, IL-1β + mannose + oe-NC or IL-1β + mannose + oe-MPI. **g** Western blotting and quantitative analysis of nuclear-cytoplasmic MondoA protein in NP cells treated with oe-NC, IL-1β + oe-NC, IL-1β + mannose + oe-NC or IL-1β + mannose + oe-MPI. Unless otherwise specified, 10 ng/ml IL-1β and 40 mmol/L mannose were used, *n* = 3. ^*^P < 0.05, ^**^P < 0.01, ^****^P < 0.0001. ns non-significant, MMP13 matrix metalloproteinase 13, IL-1β interleukin-1β, 2-DG 2-deoxy-D-glucose, TXNIP thioredoxin-interacting protein, DAPI 4’,6-diamidino-2-phenylindole, MondoA max-like protein X-interacting protein, MPI mannose phosphate isomerase

Using RT-qPCR and Western blotting, a similar trend in the effects of 2-deoxy-D-glucose (2-DG, a mannose analog) and mannose on *Mmp13* and *Txnip* (Fig. 3a, b; Additional file 1: Fig. S5a). Upon cell entry, mannose is phosphorylated into mannose 6-phosphate (M6P) [19]. Interestingly, hexose 6-phosphate can bind to the MondoA transcription factor, thereby promoting *Txnip* expression [20]. To validate whether M6P can directly interact with MondoA, molecular docking techniques were employed and it was discovered that M6P exhibits a similar binding affinity and hydrogen bond interaction site (THR101/LEU120/GLN105) to MondoA as glucose 6-phosphate (Fig. 3c; Additional file 1: Tables S3, S4). Subsequently, confocal microscopy was utilized to determine the nuclear and cytoplasmic distribution of MondoA in normal group, IL-1β group, and IL-1β + mannose group. A significant decrease in the nuclear distribution of MondoA under inflammatory stimulation was observed, while mannose effectively reversed the increase in the distribution of MondoA (Fig. 3d). MPI facilitates the metabolism of M6P into the glycolytic pathway [19]. Thus, after overexpressing MPI, a reduction in the anti-MMP effect of mannose was observed, along with a decrease in TXNIP expression, as confirmed by RT-qPCR and Western blotting (Fig. 3e, f; Additional file 1: Fig. S5b, c). Furthermore, through nuclear-cytoplasmic Western blotting analysis, a significant reduction in the expression of nuclear MondoA in response to IL-1β was noted. Notably, mannose effectively counteracted the deleterious effects of IL-1β, while the overexpression of MPI restored nuclear MondoA expression even in the presence of inflammatory conditions (Fig. 3g). Notably, throughout the aforementioned treatments, cytoplasmic MondoA expression remained unaltered.

In conclusion, it was demonstrated that mannose can directly bind to MondoA in the form of M6P, promoting the nuclear translocation of MondoA and consequently activating TXNIP expression.

### Mannose exerts anti-catabolic effects by reducing glutamine levels in vitro

**Fig. 4:**
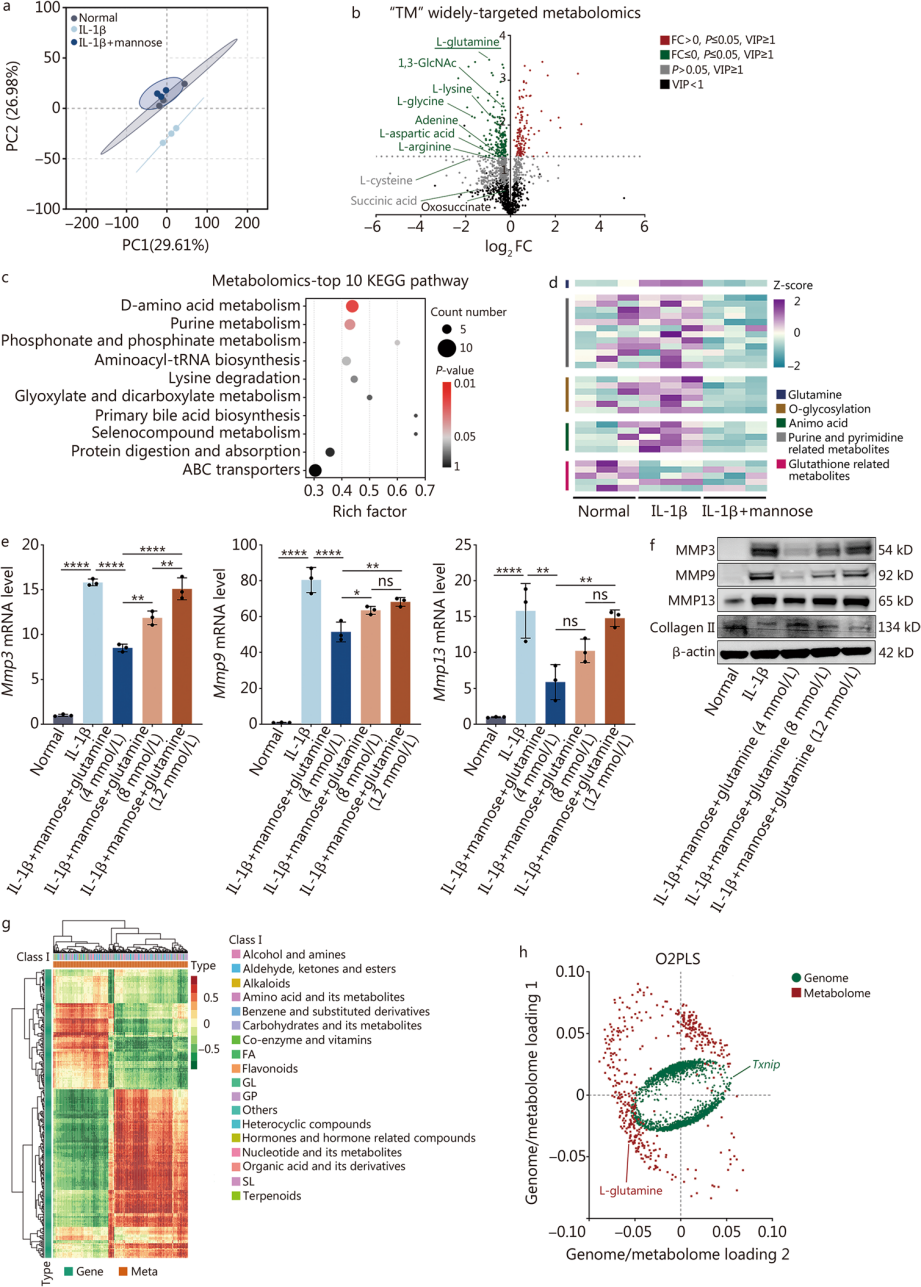
Mannose suppresses catabolism by inhibiting glutamine in vitro. **a** Principal component analysis (PCA) of “TM” widely targeted metabolomics among normal group, IL-1β group and IL-1β + mannose group. **b** Volcano plot of the metabolomic data between IL-1β group and IL-1β + mannose group. **c** Comparison of the top 10 KEGG pathways associated with metabolism between IL-1β group and IL-1β + mannose group. **d** Metabolomic heatmap of IL-1β group and IL-1β + mannose group. **e** RT-qPCR analysis of *Mmp3*, *Mmp9* and *Mmp13* in NP cells treated with IL-1β and IL-1β + different concentrations of glutamine (4 mmol/L, 8 mmol/L, or 12 mmol/L). **f** Western blotting of MMP3, MMP9, MMP13 and collagen II in NP cells treated with IL-1β and IL-1β combined with different concentrations of glutamine (4 mmol/L, 8 mmol/L, or 12 mmol/L). **g** Correlation heatmap between genes and metabolites among the three groups. **h** O2PLS model analysis between transcriptomics and metabolomics. 10 ng/ml IL-1β and 40 mmol/L mannose were used, *n* = 3. ^*^P < 0.05, ^**^P < 0.01, ^****^P < 0.0001. ns non-significant, IL-1β interleukin-1β, VIP variable importance in projection, KEGG Kyoto Encyclopedia of Genes and Genomes, ABC adenosine triphosphate-binding cassette, O2PLS two-way orthogonal PLS, FA fatty acid, GL glucose, GP plycerol phosphate, SL sphingolipid, MMP3 matrix metalloproteinase 3, MMP9 matrix metalloproteinase 9, MMP13 matrix metalloproteinase 13

As a metabolite, mannose initiates a series of reactions upon entering the cell. To further investigate the anti-MMP mechanism of mannose, “TM” widely targeted metabolomics was conducted to assess the levels of metabolites in normal group, IL-1β group, and IL-1β + mannose group. PCA of the principal components revealed a significant difference between IL-1β group and the other two groups (Fig. 4a). Moreover, PCA indicated that mannose almost completely reversed the changes in the metabolite composition induced by IL-1β in NP cells, highlighting the significant correlation between mannose and cellular metabolism (Fig. 4a). Overall, there were 186 significantly differentially expressed metabolites (VIP ≥ 1 and *P* ≤ 0.05) between normal and IL-1β groups and 332 significantly differentially expressed metabolites between the IL-1β and IL-1β + mannose groups. Among these, 108 metabolites were consistently altered in both comparisons (Additional file 1: Fig. S6a). Thus, compared with transcriptomics, the use of mannose may have a more pronounced impact on the changes in metabolites. Metabolomic analysis revealed that the amino acid metabolism pathway was the most significantly altered pathway, with L-glutamine being the most significantly altered metabolite (Fig. 4b, c). L-amino acids are widely present in nature, while D-amino acids are less common. According to metabolomic results, the presence of D-glutamine has not yet been detected. Therefore, unless otherwise specified, “glutamine” was used to refer to L-glutamine [21]. During cellular uptake, glutamine is metabolized for purine and pyrimidine synthesis, O-glycosylation metabolism, and the synthesis of other amino acids and metabolites [22]. Mannose effectively reversed the changes in the aforementioned pathways induced by IL-1β (Fig. 4c, d). Transcriptomic analysis also revealed that mannose reversed some of the IL-1β-induced changes in the expression of genes related to glutamine metabolism (Fig. 2b, c). To validate the relationship between glutamine and catabolism, in vitro experiments were conducted in which the concentration of glutamine was gradually increased (the basal concentration of glutamine in the culture medium was 4 mmol/L, and subsequently added to 8 mmol/L and 12 mmol/L). RT-qPCR and Western blotting confirmed that high concentrations of glutamine stimulate MMPs and decrease the expression of collagen II (Fig. 4e, f; Additional file 1: Fig. S6b). Without any specific notation, the subsequent experiments used a glutamine concentration of 8 mmol/L.

In summary, it was found that mannose exerts its anti-catabolic effects primarily by reducing intracellular glutamine levels.

### Mannose regulates the intracellular level of glutamine by upregulating TXNIP

**Fig. 5:**
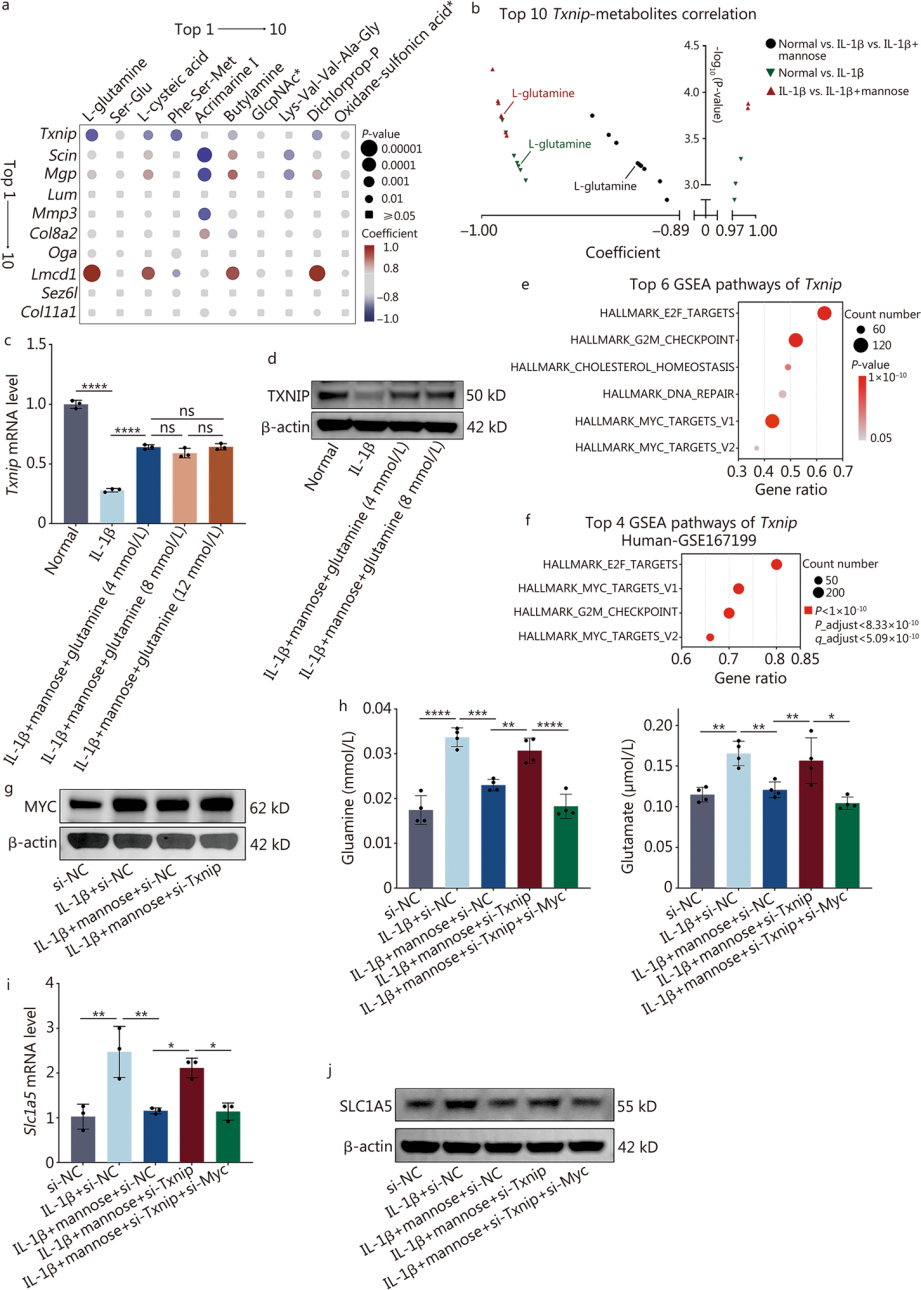
Mannose decreases the intracellular level of glutamine by upregulating TXNIP. **a** Correlation heatmap of the top 10 genes and top 10 metabolites among the three groups. GlcpNAc^*^: alpha-L-Fucp-(1- > 3)-[beta-D-Galp-(1- > 4)]-D-GlcpNAc; Oxidane-sulfonic acid^*^: [3-(5,7-dihydroxy-4-oxo-4H-chromen-2-yl)phenyl]oxidanesulfonic acid. **b** The top 10 metabolites associated with *Txnip* in different groups. RT-qPCR (**c**) and Western blotting (**d**) analysis of TXNIP in NP cells treated with IL-1β and IL-1β + different concentrations of glutamine. Top 6 GSEA pathways of *Txnip* in our transcriptomics dataset (**e**) and the top 4 GSEA pathways of *Txnip* in a human intervertebral disc degeneration tissue transcriptomics dataset (GSE167199, **f**). **g** Western blotting of MYC in NP cells treated with si-NC, IL-1β + si-NC, IL-1β + mannose + si-NC and IL-1β + mannose + si-Txnip.** h** Intracellular glutamine and glutamate levels in NP cells treated with si-NC, IL-1β + si-NC, IL-1β + mannose + si-NC, IL-1β + mannose + si-Txnip, or IL-1β + mannose + si-Txnip + si-Myc. RT-qPCR (**i**) and Western blotting (**j**) of SLC1A5 in NP cells treated with si-NC, IL-1β + si-NC, IL-1β + mannose + si-NC, IL-1β + mannose + si-Txnip, or IL-1β + mannose + si-Txnip + si-Myc. Unless otherwise specified, 10 ng/ml IL-1β and 40 mmol/L mannose were used, *n* = 3. ^*^P < 0.05, ^**^P < 0.01, ^***^P < 0.001, ^****^P < 0.0001. ns non-significant, Txnip thioredoxin-interacting protein, Scin scinderin, Mgp matrix Gla protein, Lum lumican, Mmp3 matrix metallopeptidase 3, Col8a2 collagen type VIII alpha 2 chain, Oga O-GlcNAcase, Lmcd1 LIM and cysteine-rich domains 1, Sez6l seizure-related 6 homolog-like, Col11a1 collagen type XI alpha 1 chain, IL-1β interleukin-1β, G2M gap 2 to mitosis phase, SLC1A5 solute carrier family 1 member 5

The gene-metabolite clustering heatmap revealed a strong overall correlation between genes and metabolites (Fig. 4g). Based on the integrated analysis of transcriptomics and metabolomics using the two-way orthogonal PLS (O2PLS) model, it was determined that the *Txnip* gene had the most significant impact on the metabolites, while glutamine also had some influence on the genes (Fig. 4h). This finding suggested that mannose may primarily induce changes in downstream metabolites through the *Txnip* gene. Correlation analysis between the top 10 genes and the top 10 metabolites among the three groups revealed a significant correlation between *Txnip* and glutamine, with both their coefficient values and *P*-values ranking 8/100 (Fig. 5a). Subsequently, the top 10 metabolites correlated with *Txnip* in the comparisons of normal group vs. IL-1β group, IL-1β group vs. IL-1β + mannose group, and normal group vs. IL-1β group vs. IL-1β + mannose group were analyzed. The results revealed that glutamine was the only metabolite among the top 10 correlated metabolites in all three comparisons (Fig. 5b). These analyses suggest a potential regulatory relationship between *Txnip* and glutamine. However, RT-qPCR and Western blotting analyses demonstrated that the expression of TXNIP did not significantly change with increasing levels of glutamine (Fig. 5c, d; Additional file 1: Fig. S6c). Based on the O2PLS analysis, which revealed that the *Txnip* gene had the most significant impact on the metabolome, it was hypothesized that *Txnip* might influence the intracellular level of glutamine. The Pearson correlation coefficients between *Txnip* and other genes were calculated using our own transcriptome data and the GEO dataset (human tissues-GSE167199). Subsequently, GSEA based on the genes’ correlations with *Txnip* and the GSEA results were ranked. The results showed significant differences for HALLMARK_MYC_TARGETS_V1 and HALLMARK_ MYC_TARGETS_V2, which ranked 5th and 6th, respectively, in our own transcriptome data and 2nd and 4th, respectively, in the GEO dataset (human tissues-GSE167199) (Fig. 5e, f). The 4 sets of GSEA data collectively indicated that *Txnip* suppresses downstream genes related to *Myc* transcription in IVDD (Additional file 1: Fig. S7a, b). Additionally, a trend toward enrichment of HALLMARK_MYC_TARGETS_V1 (NES = 1.38, *q* = 0.260) and HALLMARK_MYC_TARGETS_V2 (NES = 1.48, *P* = 0.071) in IL-1β group compared with IL-1β + mannose group was observed (Additional file 1: Fig. S7c, d). Lim et al. [23] discovered that TXNIP loss increases MYC-dependent gene expression by increasing *Myc* genome occupancy in several types of cells rather than by directly affecting MYC protein expression. Consistent with the findings of Lim et al. [23], it was observed that knocking down *Txnip* in NP cells did not significantly alter the expression of MYC (Fig. 5g; Additional file 1: Fig. S8a). Therefore, it is suggested that TXNIP may decrease MYC-dependent gene expression by decreasing *Myc* genome occupancy within NP cells. Previous studies have indicated a strong association between MYC and glutamine metabolism [24, 25]. MYC directly promotes the cellular uptake of glutamine by increasing the transcription of SLC1A5 (the main glutamine transporter) through the enhancer box (E-box, CACGTG) [26]. Intriguingly, Lim et al. [23] reported that MYC-dependent genes affected by TXNIP loss are highly and significantly enriched for canonical CACGTG MYC-binding E-boxes. Our transcriptomic analysis also revealed a significant upregulation of *Slc1a5* expression in response to mannose (Fig. 2b). To verify the relationships among mannose/TXNIP, MYC and SLC1A5/glutamine metabolism, the changes in intracellular glutamine, glutamate and SLC1A5 expression in *Txnip* knockdown group and the group with simultaneous knockdown of *Txnip* and *Myc* were compared (Additional file 1: Fig. S8b). *Txnip* knockdown reversed the changes in the intracellular glutamine, glutamate, and SLC1A5 levels induced by mannose treatment. Additionally, simultaneous knockdown of *Txnip* and *Myc* reversed the increase in the levels of intracellular glutamine, glutamate, and SLC1A5 caused by *Txnip* knockdown (Fig. 5h-j; Additional file 1: Fig. S8c). Based on the above results, it is believed that the hypothesis is supported, which suggests that the increase in TXNIP induced by mannose inhibits MYC-dependent transcriptional programs by increasing *Myc* genome occupancy in IVDD. To further investigate the mechanism by which mannose regulates glutamine, the impact of mannose on the glutamine efflux gene *Slc7a5* (solute carrier family 7 member 5) was studied. RT-qPCR revealed that mannose did not significantly affect the expression of *Slc7a5* (Additional file 1: Fig. S8d).

In conclusion, it was demonstrated that mannose can suppress MYC-dependent transcriptional programs through the *Txnip* gene, leading to a reduction in SLC1A5 expression and ultimately decreasing intracellular glutamine levels.

### Glutamine induces catabolism through the NH_4_^+^-mediated MAPK pathway

**Fig. 6:**
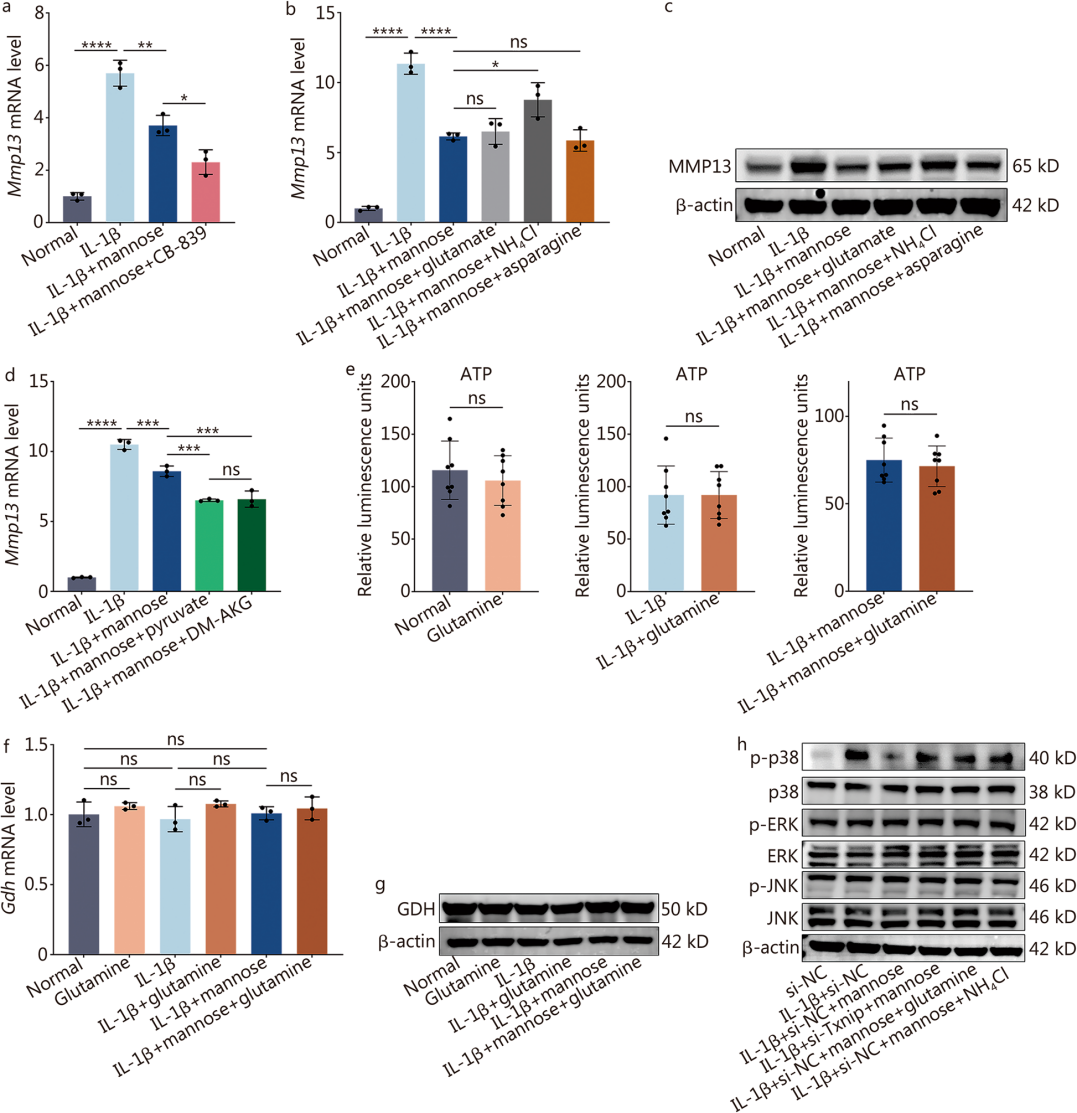
Glutamine induces catabolism through the NH_4_^+^-mediated MAPK pathway. **a** RT-qPCR analysis of *Mmp13* in NP cells treated with IL-1β, IL-1β + mannose, or IL-1β + mannose + CB-839 (1 µmol/L). RT-qPCR (**b**) and Western blotting (**c**) of MMP13 in NP cells treated with IL-1β, IL-1β + mannose, or IL-1β + mannose + glutamate (200 μmol/L)/NH_4_Cl (2 mmol/L)/asparagine (1 mmol/L). **d** RT-qPCR analysis of *Mmp13* in NP cells treated with IL-1β, IL-1β + mannose, or IL-1β + mannose + pyruvate (2 mmol/L)/DM-AKG (2 mmol/L) [dimethyl α-ketoglutarate (DM-AKG) is a cell-permeable derivative of α-ketoglutarate] [31]. **e** Intracellular ATP levels in NP cells from different groups. RT-qPCR (**f**) and Western blotting (**g**) of GDH in NP cells treated with glutamine, IL-1β, IL-1β + glutamine, IL-1β + mannose, or IL-1β + mannose + glutamine. **h** Western blotting of key MAPK pathway proteins in NP cells treated with si-NC, IL-1β + si-NC, IL-1β + mannose + si-NC, or IL-1β + mannose + si-Txnip/si-NC + glutamine/si-NC + NH_4_Cl. Unless otherwise specified, 10 ng/ml IL-1β, 40 mmol/L mannose, and 8 mmol/L glutamine were used, *n* = 3. ^*^P < 0.05, ^**^P < 0.01, ^***^P < 0.001, ^****^P < 0. 0001. ns non-significant, MMP13 matrix metalloproteinase 13, IL-1β interleukin-1β, TXNIP thioredoxin-interacting protein, DM-AKG dimethyl α-ketoglutarate, ATP adenosine triphosphate, GDH glutamate dehydrogenase 1, ERK extracellular signal-regulated kinases, JNK c-Jun N-terminal kinase

To further investigate the mechanism of glutamine-induced MMPs, CB-839, a potent and selective glutaminase inhibitor, was initially employed to simulate glutamine deprivation conditions [27, 28]. The RT-qPCR results demonstrated that *Mmp13* expression was inhibited (Fig. 6a). Glutamine is required for the biosynthesis of nonessential amino acids, and mannose primarily affects the amino acid metabolism pathway (Fig. 4c). Therefore, downstream amino acids related to glutamine were investigated. Asparagine and glutamate are the two amino acids most directly metabolized from glutamine, and in the process of generating glutamate, NH_4_^+^ is produced [22]. RT-qPCR and Western blotting revealed that glutamate and asparagine did not significantly affect the expression of MMP13, but NH_4_Cl significantly increased the expression of MMP13 (Fig. 6b, c; Additional file 1: Fig. S8e). However, several studies have shown that glutamine can support the tricarboxylic acid (TCA) cycle, thereby inhibiting inflammation [29, 30]. Therefore, IL-1β + mannose-treated NP cells were supplemented with TCA cycle key metabolites separately: pyruvate and α-ketoglutarate [dimethyl α-ketoglutarate (DM-AKG) is a cell-permeable derivative of α-ketoglutarate] [31]. RT-qPCR results indicated that the aforementioned intervention can inhibit the expression of *Mmp13* (Fig. 6d). However, KEGG, GO and GSEA analyses indicated that oxidative phosphorylation and pyruvate metabolism did not significantly differ between IL-1β and IL-1β + mannose groups (Fig. 2d; Additional file 1: Figs. S2, S9). And, in both normal NP cells, IL-1β-treated NP cells, and IL-1β + mannose-treated NP cells, supplementation with glutamine did not significantly increase intracellular ATP levels (Fig. 6e). Furthermore, RT-qPCR and Western blotting results showed that the mRNA and protein expression of GDH was not affected by glutamine supplementation (Fig. 6f, g; Additional file 1: Fig. S10a). Based on the findings, it was speculated that upon entering NP cells, glutamine may not be metabolized into α-ketoglutarate and enter the TCA cycle but rather may be metabolized into NH_4_^+^ and induce the production of MMPs. Transcriptome sequencing revealed that mannose primarily reversed IL-1β-induced PI3K, MAPK and other pathways (Fig. 2d). The PI3K pathway mainly inhibits inflammation through the nuclear factor kappa light chain enhancer of activated B cells (NF-κB) downstream pathway [32]. However, KEGG analysis revealed no significant changes in the NF-κB pathway between the IL-1β and IL-1β + mannose groups (Fig. 2d). MAPK can independently activate inflammation, separate from the NF-κB pathway [33]. Therefore, the effects of si-Txnip, glutamine, and NH_4_^+^ on the MAPK pathway were analyzed. Western blotting results demonstrated that IL-1β significantly increased the phosphorylation of p38, and mannose reversed the IL-1β-induced increase in p38 phosphorylation. However, si-Txnip, glutamine, and NH_4_^+^ all reversed the effect of mannose on p38 phosphorylation (Fig. 6h; Additional file 1: Fig. S10b).

In conclusion, mannose inhibits intracellular MMPs by reducing the levels of glutamine and NH_4_^+^ within cells, thereby acting through the MAPK pathway.

### Glutamine counteracts the ameliorating effect of mannose on IVDD in vivo

**Fig. 7:**
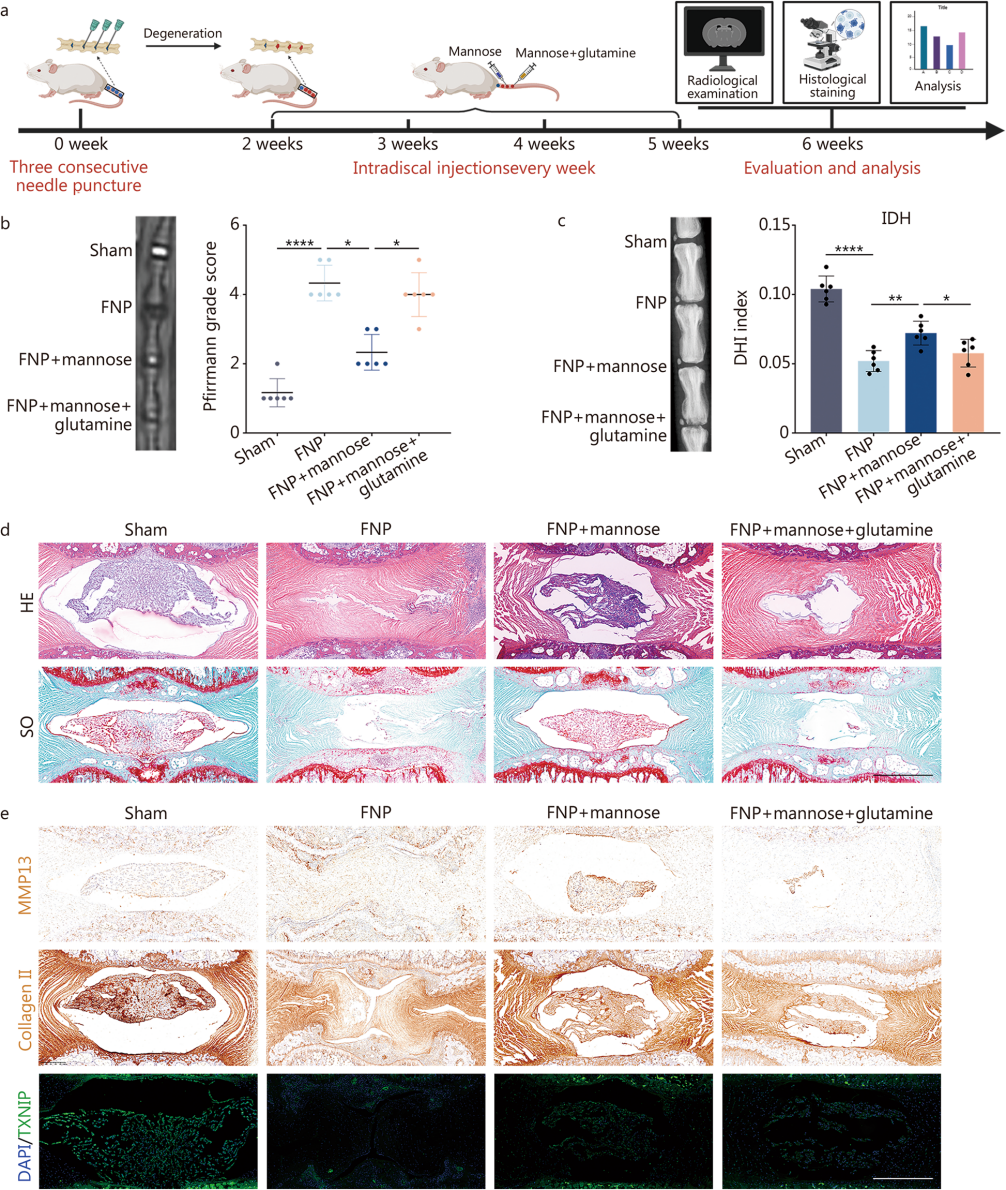
Moreover, glutamine reversed the attenuating effect of mannose on IVDD in vivo. **a** Rat intervertebral disc injection workflow diagram. **b** MR images and Pfirrmann disc degeneration grades of the sham group, FNP group, FNP + mannose group and FNP + mannose + glutamine group. **c** X-ray and IDH analyses of the sham group, FNP group, FNP + mannose group and FNP + mannose + glutamine group. **d** HE and SO staining of the sham group, FNP group, FNP + mannose group and FNP + mannose + glutamine group (original magnification × 7; scale bar = 800 µm). **e** Immunofluorescence staining of MMP13, collagen II and TXNIP in the sham group, FNP group, FNP + mannose group and FNP + mannose + glutamine group (original magnification × 7; scale bar = 800 µm). *n* = 6. ^*^P < 0.05, ^**^P < 0.01, ^****^P < 0.0001. FNP fine needle puncture, IDH intervertebral disc height, DHI disc height index, HE hematoxylin and eosin, SO safranin O, MMP13 matrix metalloproteinase 13, DAPI 4’,6-diamidino-2-phenylindole, TXNIP thioredoxin-interacting protein

As shown in Fig. 7a, mannose and glutamine were injected into the intervertebral disc to assess their effects in the animal model. The Pfirrmann disc degeneration grade and intervertebral disc height (IDH) were measured as follows: FNP successfully induced IVDD, mannose significantly alleviated IVDD, and glutamine effectively reversed the effect of mannose (Fig. 7b, c). The above results are consistent with the outcomes of the in vitro experiments. HE and SO staining were also conducted, revealing that mannose significantly rescued the amount of gelatinous NP tissue, while glutamine significantly decreased the amount of gelatinous NP tissue (Fig. 7d; Additional file 1: Fig. S11a). Subsequently, IHC for MMP13 and collagen II was performed, and the results were consistent with those of the in vitro experiments. Mannose notably reduced the expression of MMP13 in the IVDD model and promoted the expression of collagen II. However, glutamine markedly reversed the effects of mannose (Fig. 7e; Additional file 1: Fig. S11b). The IHC results also revealed the following observations: FNP induced a significant decrease in TXNIP expression; mannose significantly elevated TXNIP expression under FNP treatment, whereas glutamine treatment did not significantly alter TXNIP expression (Fig. 7e; Additional file 1: Fig. S11b).

In conclusion, the in vivo intervertebral disc injection experiments revealed that mannose reversed IVDD and that glutamine inhibited the rescue effect of mannose.

### Oral administration of mannose also rescues IVDD in vivo

**Fig. 8:**
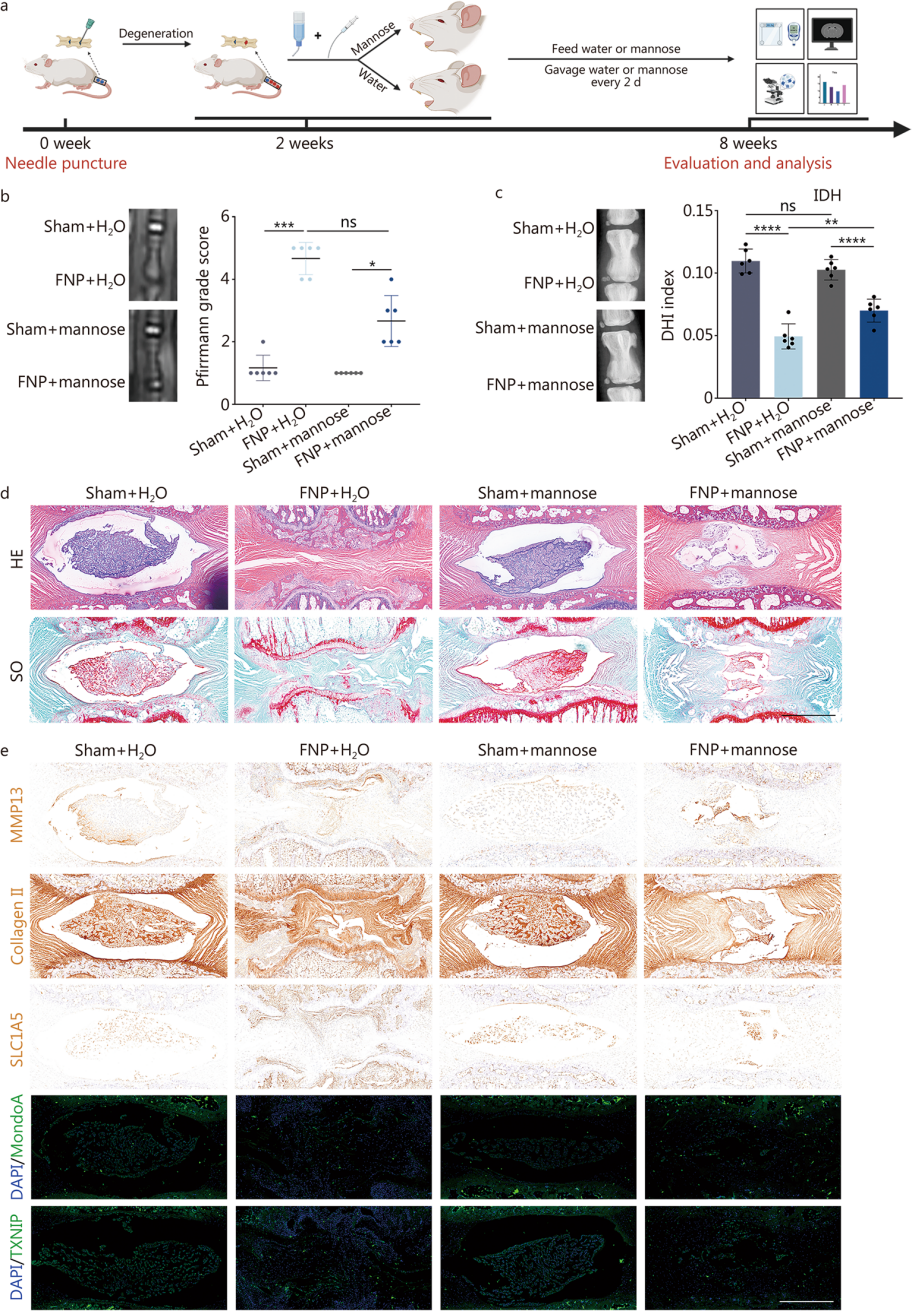
IVDD can be reversed by the oral administration of mannose. **a** Rat oral feeding workflow diagram. **b** MR images and Pfirrmann disc degeneration grades of the sham + H_2_O group, FNP + H_2_O group, sham + mannose group, and FNP + mannose group. **c** X-ray and IDH analyses of the sham + H_2_O group, FNP + H_2_O group, sham + mannose group, and FNP + mannose group. **d** HE and SO staining of the sham + H_2_O group, FNP + H_2_O group, sham + mannose group, and FNP + mannose group (original magnification × 7; scale bar = 800 µm). **e** Immunofluorescence staining of MMP13, collagen II, SLC1A5, MondoA and TXNIP in the sham + H_2_O group, FNP + H_2_O group, sham + mannose group, and FNP + mannose group (original magnification × 7; scale bar = 800 µm). Unless otherwise specified, *n* = 6. ^*^P < 0.05, ^**^P < 0.01, ^***^P < 0.001, ^****^P < 0.0001. ns non-significant, FNP fine needle puncture, IDH intervertebral disc height, DHI disc height index, HE hematoxylin and eosin, SO safranin O, MMP13 matrix metalloproteinase 13, SLC1A5 solute carrier family 1 member 5, DAPI 4’,6-diamidino-2-phenylindole, MondoA max-like protein X-interacting protein, TXNIP thioredoxin-interacting protein

Mannose, an oral medication, has been clinically used to treat urinary tract infections [34]. To verify the oral efficacy of mannose in IVDD treatment, mannose or water was administered through oral gavage, as depicted in Fig. 8a. No significant difference in body weight was observed between the group receiving regular drinking water and the group receiving oral mannose solution over a 6-week period (Additional file 1: Fig. S11c). Additionally, no significant changes in blood sugar were observed at the end of the 6-week period (Additional file 1: Fig. S11d). The above results indicated that this treatment concentration has no significant adverse reaction. Then, the Pfirrmann disc degeneration grade and IDH were assessed. It was found that oral administration of mannose had no significant effect on normal intervertebral discs but did significantly alleviate IVDD (Fig. 8b, c). Similarly, HE and SO staining experiments revealed that oral mannose administration rescued IVDD (Fig. 8d; Additional file 1: Fig. S11e). According to the IHC results, mirroring the trend observed in mannose intervertebral disc injection experiments, oral mannose administration significantly reversed the effects of FNP on the expression of MMP13, Collagen II, SLC1A5, MondoA transcription factor, and TXNIP (Fig. 8e; Additional file 1: Fig. S11f).

In conclusion, oral administration of mannose can ameliorate IVDD by inhibiting glutamine intake without causing significant systemic toxic effects.

## Discussion

Previous studies have indicated that mannose has anti-inflammatory effects on various diseases, such as asthma, colitis and osteoarthritis [35]. These findings suggest that the administration of mannose could be a potential treatment approach for multiple diseases simultaneously. Notably, there is a lack of published research on the preventive effects of mannose on inflammatory diseases. Investigating both aspects mentioned above will further expand the clinical applications of mannose. Although many studies have employed mannose concentrations lower than 40 mmol/L, our CCK-8 experiments revealed that NP cells have a maximum tolerance to mannose at a concentration of 40 mmol/L [19, 35]. Therefore, a more precise exploration of the dosing of orally administered mannose is crucial for its clinical application.

This study demonstrated that mannose can directly target MondoA, thereby abrogating the decrease in TXNIP expression in IVDD patients and ultimately alleviating IVDD. However, several studies have demonstrated an increase in TXNIP levels in NP cells under inflammatory stimulation [36, 37]. This study revealed that TXNIP expression decreases under inflammatory stimulation both in vivo and in vitro, which is consistent with the findings of other studies [38–40]. Additionally, the analysis of human intervertebral disc tissue, human/rat/mouse osteoarthritis chondrocyte tissue and human osteoarthritis synovial tissue from published data and the GEO database also revealed a decrease in *Txnip* expression under inflammatory conditions [18]. The discrepancy in findings might be attributed to some studies using H_2_O_2_ as an inflammatory stimulus. Furthermore, our transcriptomic results indicate that mannose most significantly affects the expression of *Txnip*. In addition, various techniques, including molecular docking, confocal microscopy, and nucleocytoplasmic protein analysis were employed in this study to substantiate the direct regulatory relationship between mannose and TXNIP. Notably, knockdown of *Txnip* significantly attenuated the anti-MMP effect of mannose. Overall, identification of one of the main pathways through which mannose exerts its effects has been achieved.

Currently, research suggests that both excessive and decreased levels of TXNIP can lead to various cellular disease states [41–43]. In our experiments, although mannose significantly increased cellular TXNIP expression under inflammatory stimulation both in vivo and in vitro, the maximum concentration of mannose did not increase TXNIP levels in IL-1β-induced NP cells beyond those in normal NP cells. This may be attributed to the self-adaptive regulation of the MPI within NP cells [19]. Moreover, oral administration of mannose did not significantly increase the levels of MondoA or TXNIP in normal intervertebral disc tissues in vivo. However, it notably increased the levels of TXNIP within degenerated tissues. This may be attributed to the potential sharing of glucose transporters by mannose, and under conditions of IVDD, there is a significant increase in sugar uptake by degenerated tissues [44]. Surprisingly, upon entering the cell, not only mannose but also other hexoses undergo phosphorylation to form hexose 6-phosphates and induce TXNIP. It has been demonstrated that mannose, D-allose and 2-DG have anti-inflammatory effects, while D-fructose, D-glucose and D-galactose exert proinflammatory effects [45–49]. For the aforementioned reasons, existing studies have demonstrated that D-allose 6-phosphate and 2-DG 6-phosphate cannot be further metabolized and enter glycolysis within the cell [50, 51]. Due to variations in intracellular MPI activity, M6P can undergo slow metabolism in certain cells, thereby exerting anti-inflammatory effects [18]. However, D-fructose, D-glucose, and D-galactose can easily be metabolized to the glycolysis/TCA cycle. Therefore, conducting thorough research on the relationship between hexoses and TXNIP/energy metabolism will further help us understand their anti-inflammatory effects.

Additionally, it was observed that mannose alleviated IVDD by reducing intracellular glutamine levels in NP cells. Glutamine has been recognized as a crucial amino acid that provides both carbon and nitrogen to support various biosynthetic processes [22]. Consistent with our findings, previous study has shown that IL-1β can accelerate the cellular uptake and utilization of glutamine [52]. This study also revealed that mannose significantly abrogated the increase in the expression of the glutamine transporter SLC1A5 both in vitro and in vivo. However, it did not significantly affect the efflux transporter gene of *Slc7a5*. The metabolism of glutamine to glutamate leads to the production of NH_4_^+^. Previous studies have shown that NH_4_^+^ promotes apoptosis and inflammation [53, 54], and this study provides the first evidence that NH_4_^+^ induces MMPs in NP cells via the MAPK pathway. However, glutamine can be metabolized into α-ketoglutarate and enter the TCA cycle within the cell. Some studies have suggested that α-ketoglutarate has anti-inflammatory effects [30, 55]. This study also confirmed that the TCA cycle metabolites pyruvate and α-ketoglutarate can suppress MMPs. Interestingly, NP cells do not preferentially utilize glutamine as an energy substrate, which is consistent with the findings of Johnston et al. [56]. In summary, within NP cells, glutamine does not primarily enter the TCA cycle to achieve an anti-inflammatory effect. Therefore, mannose can reduce the generation of NH_4_^+^ resulting from glutamine degradation, thereby inhibiting NH_4_^+^-induced MAPK activation and consequently suppressing MMPs.

There may be a regulatory relationship between TXNIP and glutamine metabolism. Kaadige et al. [57] suggested that glutamine inhibits TXNIP expression by influencing the TCA cycle. However, this study found that supplementation of NP cells with glutamine did not suppress TXNIP expression and that intravertebral disc injection of glutamine did not affect the expression of TXNIP in vivo. This disparity might stem from the reasons that this study previously mentioned: NP cells do not predominantly utilize glutamine as a substrate for the TCA cycle. Interestingly, TXNIP plays a crucial role in regulating cell metabolism [13]. An animal model in which *Txnip* is knocked down exhibited disruptions in fundamental metabolic pathways such as glucose and fatty acid metabolism [58]. In rare genetic cases of human subjects lacking TXNIP, disturbances in glucose and amino acid metabolism have been observed [14]. To further explore the relationship between TXNIP and glutamine, a GSEA of genes associated with *Txnip* was conducted. The RNA sequencing results and human tissue sequencing results revealed the most significant negative correlations between E2F and MYC-related genes and *Txnip* in this study. Additionally, both E2F and MYC act as transcription factors for SLC1A5. LIM and cysteine-rich domain 1 (LMCD1) acts as a transcriptional coactivator of E2F and exhibits a strong significant correlation with glutamine (Fig. 5a) [59]. In this study, focus was placed on *Myc*, the primary gene of interest, and found that mannose does not affect MYC protein expression but directly influences the levels of SLC1A5 and intracellular glutamine. Lim et al. [23] reported that knocking down *Txnip* does not affect MYC protein levels but that intracellular *Txnip* knockdown can increase MYC genomic binding in various cell types. Therefore, it is believed that mannose upregulates the *Txnip* gene, which reduces MYC genomic binding, leading to the downregulation of SLC1A5 and consequently affecting intracellular glutamine levels.

In this study, a significant decrease in TXNIP expression induced by needle puncture of rat caudal vertebrae was observed, consistent with the marked downward trend in *Txnip* expression during IVDD in humans (Fig. 2i, j). Although the rat caudal puncture model has been extensively used for studying IVDD, notable differences exist between rat and human intervertebral discs [16]. From a cellular composition perspective, although notochordal cells are the second-largest cell type in both rat and human NP after chondrocyte-like cells, the proportion of notochord-like cells in rat NP is notably greater than that in human NP [60]. One study confirmed that notochordal cells gradually decrease with age [61]. Additionally, most studies utilize young rats for modeling, whereas IVDD primarily affects older individuals [16]. This further contributes to the disparities in cellular composition between human IVDD and rat IVDD models. Notochordal cells have also been shown to play a role in promoting anabolism and resisting catabolism [61]. The varying proportions of notochordal cells between rats and humans may lead to divergent responses of rat and human NP tissues to external stimuli. Additionally, differences exist in the mechanical loading and morphology of both lumbar and caudal vertebrae and intervertebral discs, especially considering the variations in walking patterns between rats and humans [15]. Moreover, there might be significant differences in gene expression between rat and human intervertebral disc cells, potentially impacting cellular functionality and responses to external stimuli [62]. Consequently, in light of the identified limitations associated with the use of rat models, we will proceed with caution in our subsequent studies concerning the potential clinical use of mannose for treating IVDD.

## Conclusions

**Fig. 9:**
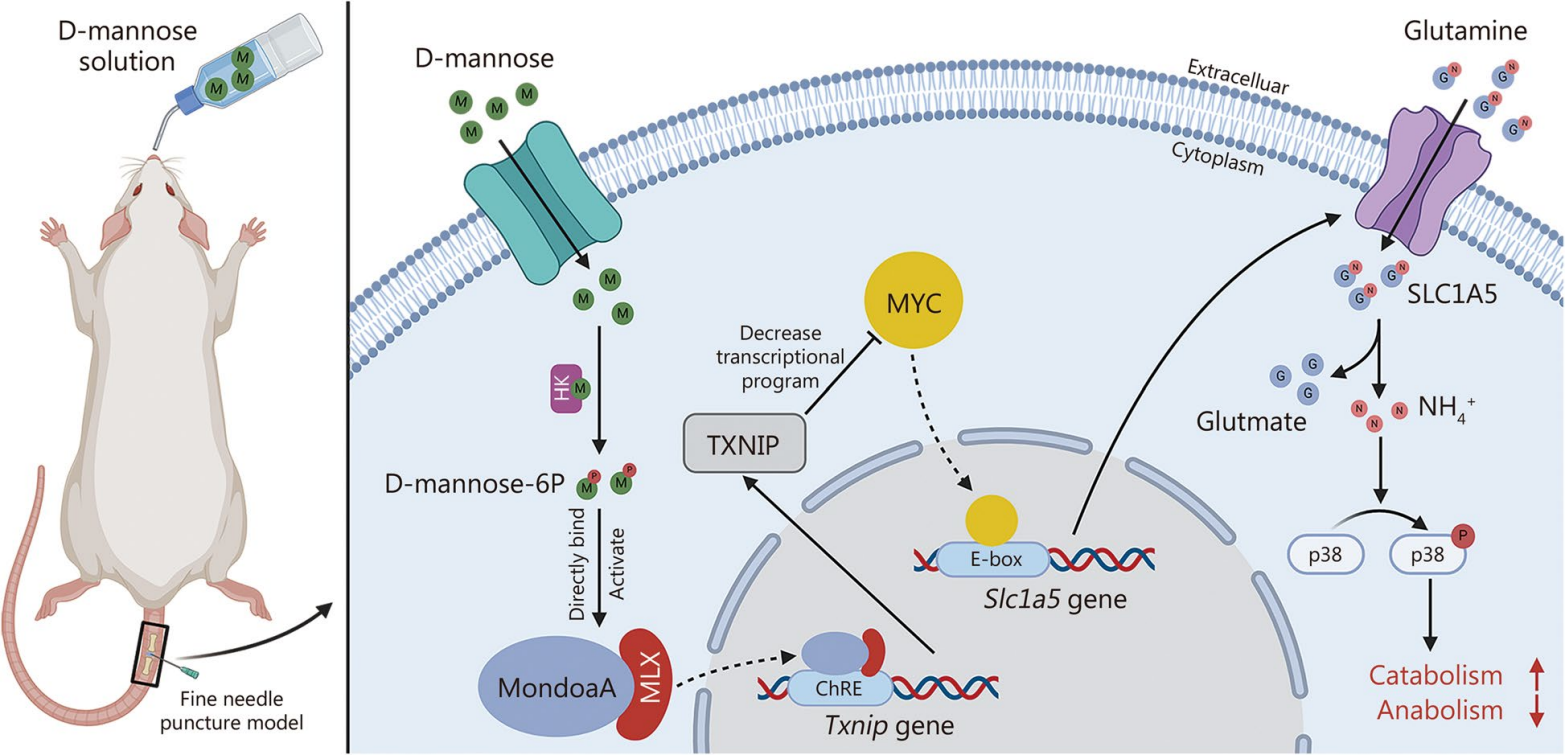
Schematic diagram of the alleviation of IVDD by mannose. HK hexokinase, MondoA max-like protein X-interacting protein, MLX max-like protein X, ChRE carbohydrate responsive element, TXNIP thioredoxin-interacting protein, E-box enhancer box, SLC1A5 solute carrier family 1 member 5

This study represents a groundbreaking achievement by demonstrating for the first time the ability of mannose to alleviate IVDD through a series of in vitro and in vivo experiments. Notably, the oral administration of mannose at the requisite concentration successfully alleviated IVDD in rat models, suggesting the potential for simplifying treatment and enhancing patient compliance in clinical applications. Furthermore, using integrated multiomics analysis techniques, the intricate mechanism underlying the effectiveness of mannose in treating IVDD was elucidated. This study provides a novel understanding that mannose directly targets the transcription factor MondoA via M6P, resulting in an increase in TXNIP levels. The elevated TXNIP levels, in turn, led to a reduction in MYC genomic binding, subsequently downregulating SLC1A5 and intracellular glutamine. This, in turn, reduces the activation of the MAPK pathway induced by NH_4_^+^ and ultimately reverses IVDD through the suppression of catabolic processes (as depicted in Fig. 9). In summary, the use of mannose in the treatment of IVDD has exceptional clinical utility, indicating that it is a highly valuable therapeutic option.

### Supplementary Information

**Additional file 1: Fig. S1** Western blotting quantitative analysis of MMP1, MMP3, MMP9, MMP13 and collagen II in NP cells treated with different concentrations of mannose (in Fig. 1e, *n* = 3). **Fig. S2** Venn diagram and GO analysis of transcriptomics. **Fig. S3** Verification of TXNIP’s small interfering RNA (si-Txnip). **Fig. S4** Gene Expression Omnibus (GEO) databases reveal that *Txnip* is significantly downregulated in human osteoarthritis synovial tissue and rat/mouse osteoarthritis chondrocyte tissue. **Fig. S5 **Western blotting quantitative analysis of **Fig. 3** and verification of overexpression (oe) plasmids. **Fig. S6** Venn diagram of “TM” widely-targeted metabolomics and Western blotting quantitative analysis of **Fig. 4f** and **Fig. 5d.** **Fig. S7** GSEA analysis. **Fig. S8** Western blotting quantitative analysis (**Figs. 5g, j** and **6c**), verification of MYC’s small interfering RNA (si-Txnip), and mRNA expression of *Slc7a5*. **Fig. S9** GSEA analysis of HALLMARK_OXIDATIVE_ PHOSPHORYLATION between the IL-1β group vs. IL-1β + mannose group. **Fig. S10** Western blotting quantitative analysis of **Fig. 6g-h**. **Fig. S11** Quantitative analysis of **Figs. 7–8**, and body weight and blood sugar of water-fed group and mannose-fed group. **Table S1** Primer sequences for RT-qPCR (5’ to 3’). **Table S2** Primer sequences of small interfering RNA (5’ to 3’). **Table S3** Interaction forces of MondoA and glucose 6-phosphate. **Table S4** Interaction forces of MondoA and mannose 6-phosphate.

## Data Availability

The datasets used or analyzed during the current study are available from the corresponding author upon reasonable request.

## Abbreviations

AMPK: AMP-activated protein kinase
Adamts4: A disintegrin and metalloproteinase with thrombospondin motifs 4
ATP: Adenosine triphosphate
ABC: Adenosine triphosphate-binding cassette
CCK-8: Cell counting kit-8
CV: Coefficient of variation
Col2a1: Collagen type II alpha 1 chain
Col8a2: Collagen type VIII alpha 2 chain
Col11a1: Collagen type XI alpha 1 chain
DEGs: Differentially expressed genes
DHI: Disc height index
DM-AKG: Dimethyl α-ketoglutarate
DAPI: 4’,6-Diamidino-2-phenylindole
EdU: 5-Ethynyl-2’-deoxyuridine
E-box: Enhancer box
ERK: Extracellular signal-regulated kinases
FDR: False discovery rate
FA: Fatty acid
FNP: Fine needle puncture
FITC: Fluorescein isothiocyanate
FC: Fold change
GSEA: Gene set enrichment analysis
GEO: Gene expression omnibus
GLUD1: Glutamate dehydrogenase 1 (also known as GDH)
GLS: Glutaminase
Gls2: Glutaminase 2
G2M: Gap 2 to mitosis phase
HE: Hematoxylin and eosin
HK: Hexokinase
IHC: Immunohistochemistry
IL-1β: Interleukin-1β
IVDD: Intervertebral disc degeneration
JAK-STAT: Janus kinase—signal transducer and activator of transcription
JNK: C-jun N-terminal kinase
LMCD1: LIM and cysteine-rich domains 1
Lum: Lumican
mTOR: Mammalian target of rapamycin
M6P: Mannose 6-phosphate
MPI: Mannose phosphate isomerase
MMP: Matrix metalloproteinase
MondoA: Max-like protein X-interacting protein (also known as MLXIP)
MAPK: Mitogen-activated protein kinase
NP: Nucleus pulposus
Oga: O-GlcNAcase
PCA: Principal component analysis
Ras: Rat sarcoma protein
SO: Safranin O
Scin: Scinderin
Sez6l: Seizure related 6 homolog like
siRNA: Small interfering RNA
SLC1A5: Solute carrier family 1 member 5
SLC7A5: Solute carrier family 7 member 5
SL: Sphingolipid
TXNIP: Thioredoxin-interacting protein
TNF: Tumor necrosis factor
t-SNE: t-distributed stochastic neighbor embedding
TCA: Tricarboxylic acid
TBS: Tris-buffered saline
UHPLC-MS: Ultra-high performance liquid chromatography-mass spectrometry
UMAP: Uniform manifold approximation and projection
VIP: Variable importance in projection
WNT: Wingless/integrated
