# Supplementary material for: D-mannose alleviates intervertebral disc degeneration through glutamine metabolism
Source: Mil Med Res. 2024 May 6;11:28. doi: 10.1186/s40779-024-00529-4 (PMC11071241; doi:10.1186/s40779-024-00529-4)
Supplement: Supplementary file 1 — Additional file 1: Fig. S1 Western blotting quantitative analysis of MMP1, MMP3, MMP9, MMP13 and collagen II in NP cells treated with different concentrations of mannose (in Fig. 1e, n = 3). Fig. S2 Venn diagram and GO analysis of transcriptomics. Fig. S3 Verification of TXNIP’s small interfering RNA (si-Txnip). Fig. S4 Gene Expression Omnibus (GEO) databases reveal that Txnip is significantly downregulated in human osteoarthritis synovial tissue and rat/mouse osteoarthritis chondrocyte tissue. Fig. S5 Western blotting quantitative analysis of Fig. 3 and verification of overexpression (oe) plasmids. Fig. S6 Venn diagram of “TM” widely-targeted metabolomics and Western blotting quantitative analysis of Fig. 4f and Fig. 5d. Fig. S7 GSEA analysis. Fig. S8 Western blotting quantitative analysis (Figs. 5g, j and 6c), verification of MYC’s small interfering RNA (si-Txnip), and mRNA expression of Slc7a5. Fig. S9 GSEA analysis of HALLMARK_OXIDATIVE_ PHOSPHORYLATION between the IL-1β group vs. IL-1β + mannose group. Fig. S10 Western blotting quantitative analysis of Fig. 6g-h. Fig. S11 Quantitative analysis of Figs. 7–8, and body weight and blood sugar of water-fed group and mannose-fed group. Table S1 Primer sequences for RT-qPCR (5’ to 3’). Table S2 Primer sequences of small interfering RNA (5’ to 3’). Table S3 Interaction forces of MondoA and glucose 6-phosphate. Table S4 Interaction forces of MondoA and mannose 6-phosphate. [file 40779_2024_529_MOESM1_ESM.pdf]

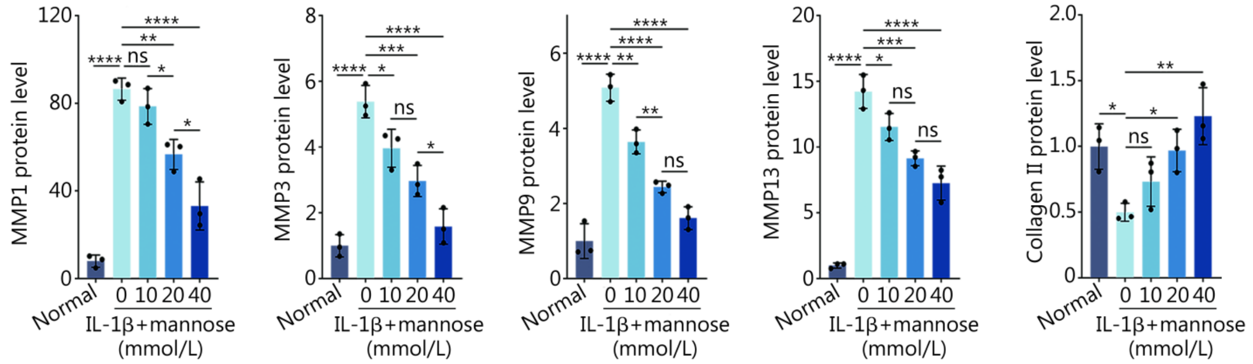

Fig. S1 Western blotting quantitative analysis of MMP1, MMP3, MMP9, MMP13 and collagen II in NP cells treated with different concentrations of mannose (in **Fig. 1e,** $n = 3$). $^{*}P < 0.05$, $^{**}P < 0.01$, $^{***}P < 0.001$, $^{****}P < 0.0001$. ns non-significant, IL-1β interleukin-1β, MMP1 matrix metalloproteinase 1, MMP3 matrix metalloproteinase 3, MMP9 matrix metalloproteinase 9, MMP13 matrix metalloproteinase 13, NP nucleus pulposus

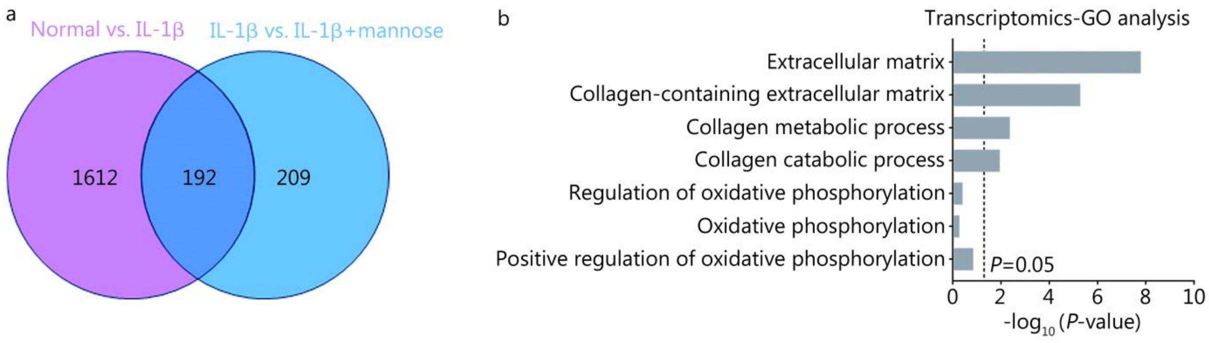

Fig. S2 Venn diagram and GO analysis of transcriptomics. **a** Differential expressed genes (DEGs) of normal group vs. IL-1β group and IL-1β group vs. IL-1β + mannose group were shown by Venn diagram. **b** Transcriptomics GO analysis between IL-1β group and IL-1β + mannose group. GO Gene Ontology, IL-1β interlenkin-1β

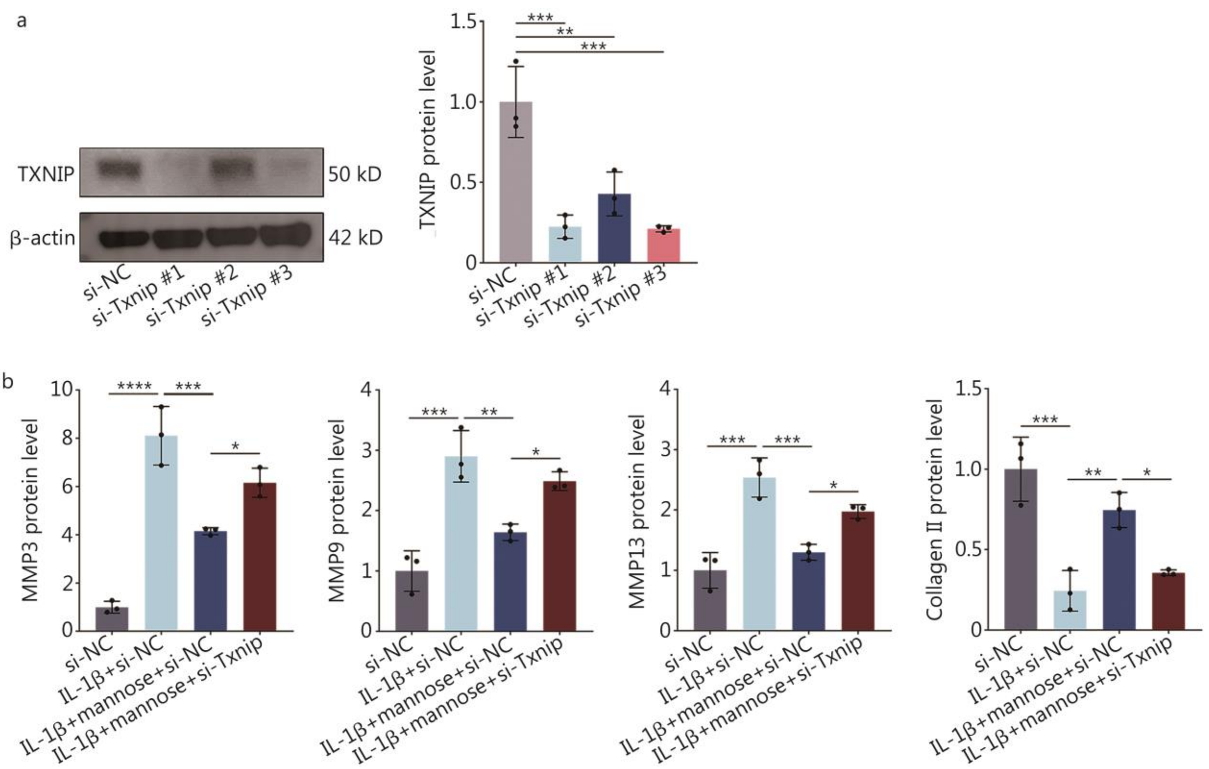

Fig. S3 Verification of TXNIP's small interfering RNA (si-Txnip). **a** Western blotting and quantitative analysis of TXNIP in NP cells treated with si-NC and three different sequences of si-Txnip. si-Txnip #3 was chosen for the following experiments. **b** Western blotting quantitative analysis of MMP3, MMP9, MMP13 and collagen II in NP cells treated with si-NC, IL-1β + si-NC, IL-1β + mannose + si-NC and IL-1β + mannose + si-Txnip (in **Fig. 2h**). 10 ng/ml IL-1β and 40 mmol/L mannose were used, $n = 3$. $^{*}P < 0.05$, $^{**}P < 0.01$, $^{***}P < 0.001$, $^{****}P < 0.0001$. IL-1β interleukin-1β, TXNIP thioredoxin-interacting protein, MMP3 matrix metalloproteinase 3, MMP9 matrix metalloproteinase 9, MMP13 matrix metalloproteinase 13, NP nucleus pulposus

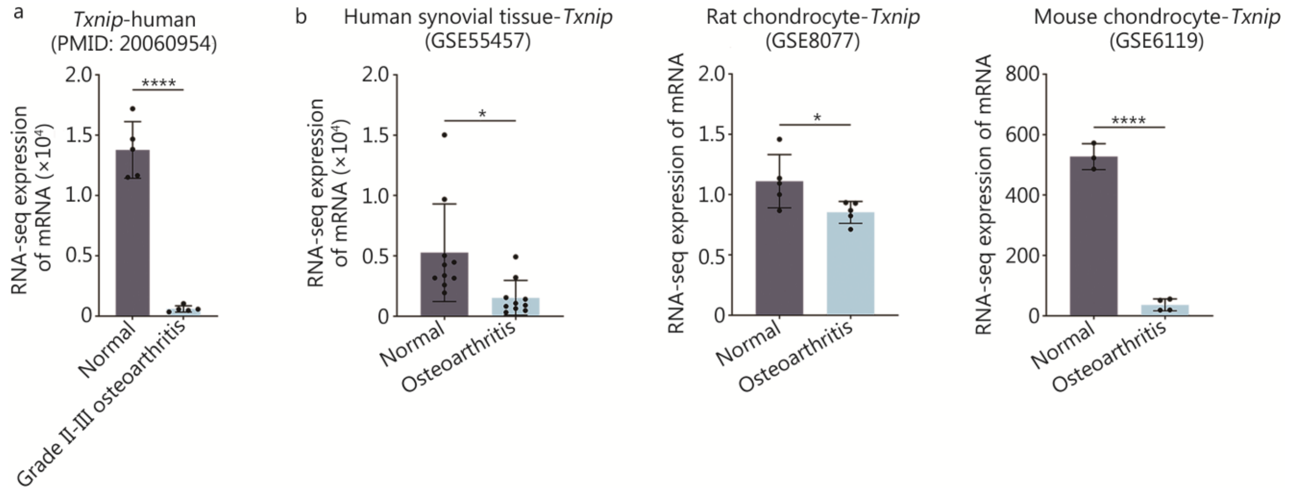

Fig. S4 Gene Expression Omnibus (GEO) databases reveal that *Txnip* is significantly downregulated in human osteoarthritis synovial tissue and rat/mouse osteoarthritis chondrocyte tissue. **a** The difference in the expression of *Txnip* between human normal chondrocyte tissues and human Grade II – III osteoarthritis chondrocyte tissues (PMID: 20060954, $n = 5$) [17]. **b** *Txnip* expression in GEO data of human osteoarthritis synovial tissue (GSE55457, $n = 10$), rat osteoarthritis chondrocyte tissue (GSE8077, $n = 5$), and mouse osteoarthritis chondrocyte tissue (GSE6119, $n = 3$ vs. $n = 4$). $^{*}P < 0.05$, $^{****}P < 0.0001$. TXNIP thioredoxin-interacting protein, RNA-seq RNA sequencing

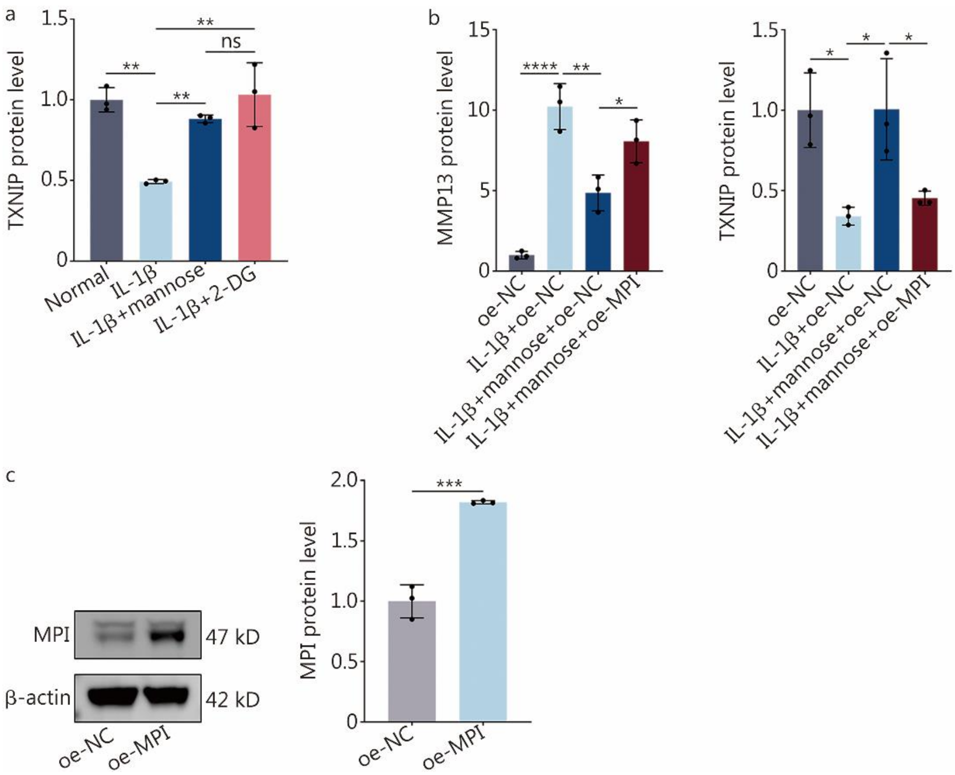

Fig. S5 Western blotting quantitative analysis of **Fig. 3** and verification of overexpression (oe) plasmids. **a** Western blotting quantitative analysis of TXNIP in NP cells treated with IL-1β, IL-1β + mannose and IL-1β + 2-DG (2 mmol/L) (in **Fig. 3b**). **b** Western blotting quantitative analysis of MMP13 and TXNIP in NP cells treated with oe-NC, IL-1β + oe-NC, IL-1β + mannose + oe-NC and IL-1β + mannose + oe-MPI (in **Fig. 3f**). **c** Western blotting and quantitative analysis of MPI in NP cells treated with oe-NC and overexpression plasmids of MPI (oe-MPI). 10 ng/ml IL-1β and 40 mmol/L mannose were used, $n = 3$. $^{*}P < 0.05$, $^{**}P < 0.01$, $^{***}P < 0.001$, $^{****}P < 0.0001$. ns non-significant, IL-1β interleukin-1β, TXNIP thioredoxin-interacting protein, MMP13 matrix metalloproteinase 13, 2-DG 2-deoxy-D-glucose, MPI mannose phosphate isomerase, NP nucleus pulposus

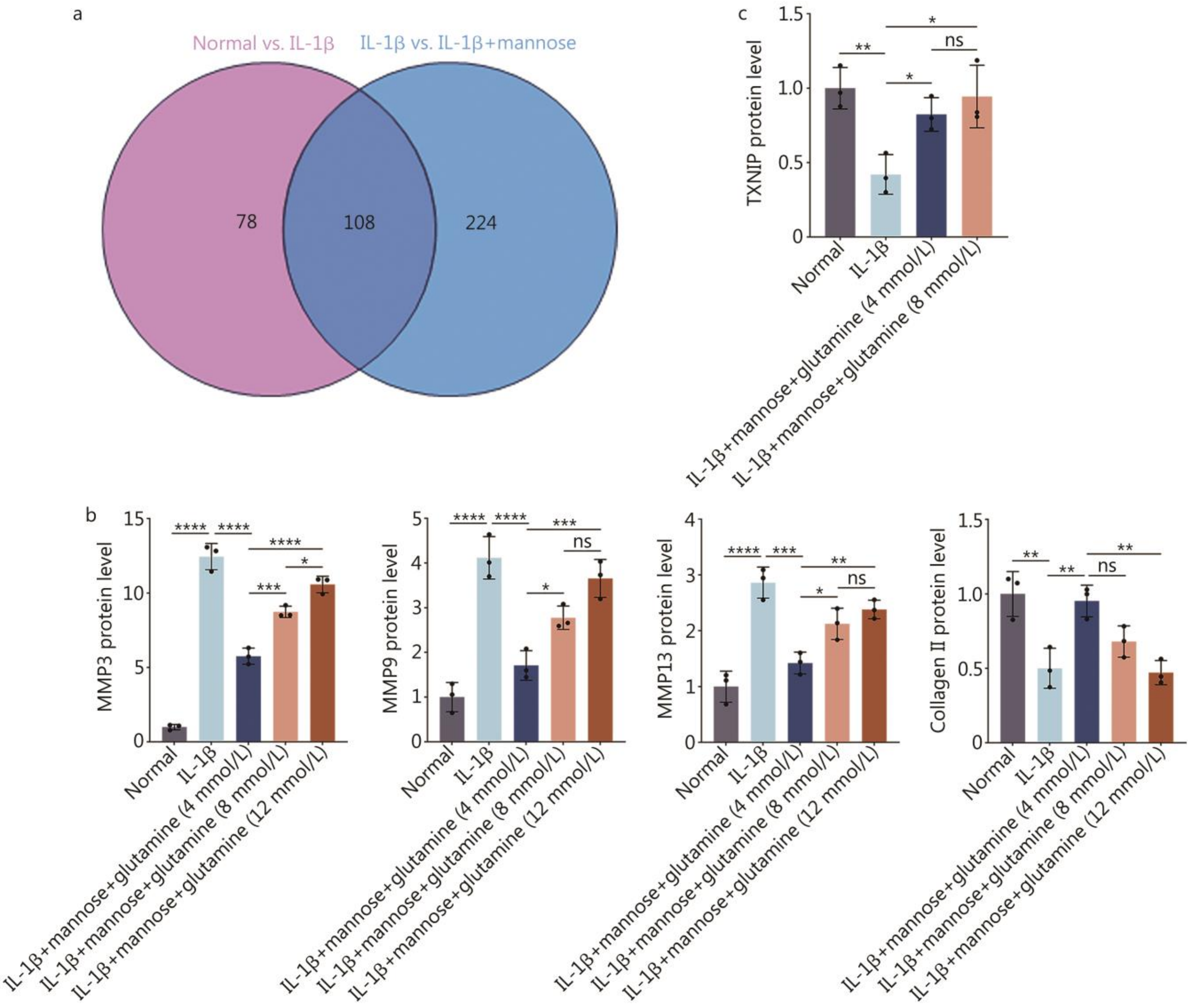

Fig. S6 Venn diagram of "TM" widely-targeted metabolomics and Western blotting quantitative analysis of **Fig. 4f** and **Fig. 5d**. **a** Venn diagram of "TM" widely-targeted metabolomics. Differentially metabolites of the normal group vs. IL-1β group and the IL-1β group vs. IL-1β + mannose group were shown by Venn diagram. IL-1β interlenkin-1β. **b** Western blotting quantitative analysis of MMP3, MMP9, MMP13 and collagen II in NP cells treated with IL-1β and IL-1β + different concentrations of glutamine (4 mmol/L, 8 mmol/L, 12 mmol/L; in **Fig. 4f**). **c** Western blotting quantitative analysis of TXNIP in NP cells treated with IL-1β and IL-1β + different concentrations of glutamine (in **Fig. 5d**). 10 ng/ml IL-1β and 40 mmol/L mannose were used, $n = 3$. $^{*}P < 0.05$, $^{**}P < 0.01$, $^{***}P < 0.001$, $^{****}P < 0.0001$. ns non-significant, IL-1β interleukin-1β, MMP3 matrix metalloproteinase 3, MMP9 matrix metalloproteinase 9, MMP13 matrix metalloproteinase 13, TXNIP thioredoxin-interacting protein, NP nucleus pulposus

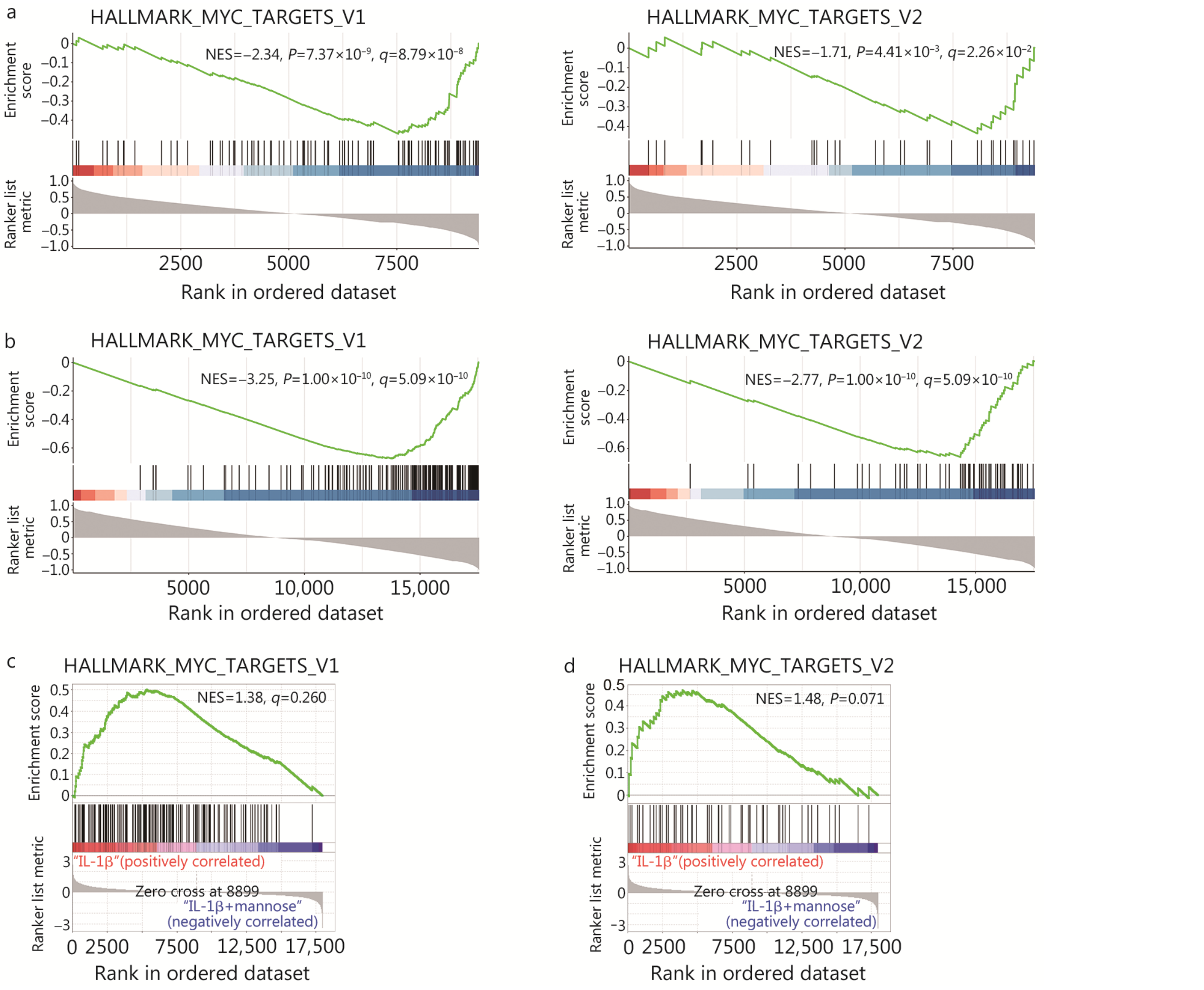

Fig. S7 GSEA analysis. **a** GSEA analysis of HALLMARK_MYC_TARGETS_V1 and HALLMARK_MYC_TARGETS_V2 in our transcriptomics. **b** GSEA analysis of HALLMARK_MYC_TARGETS_V1 and HALLMARK_MYC_TARGETS_V2 in human intervertebral disc degeneration tissue transcriptomics (GSE167199). **c** GSEA analysis of HALLMARK_MYC_TARGETS_V1 between the IL-1β group vs. IL-1β + mannose group. **d** GSEA analysis of HALLMARK_MYC_TARGETS_V2 between the IL-1β group vs. IL-1β + mannose group. GSEA gene set enrichment analysis, IL-1β interleukin-1β, NES normalized enrichment score

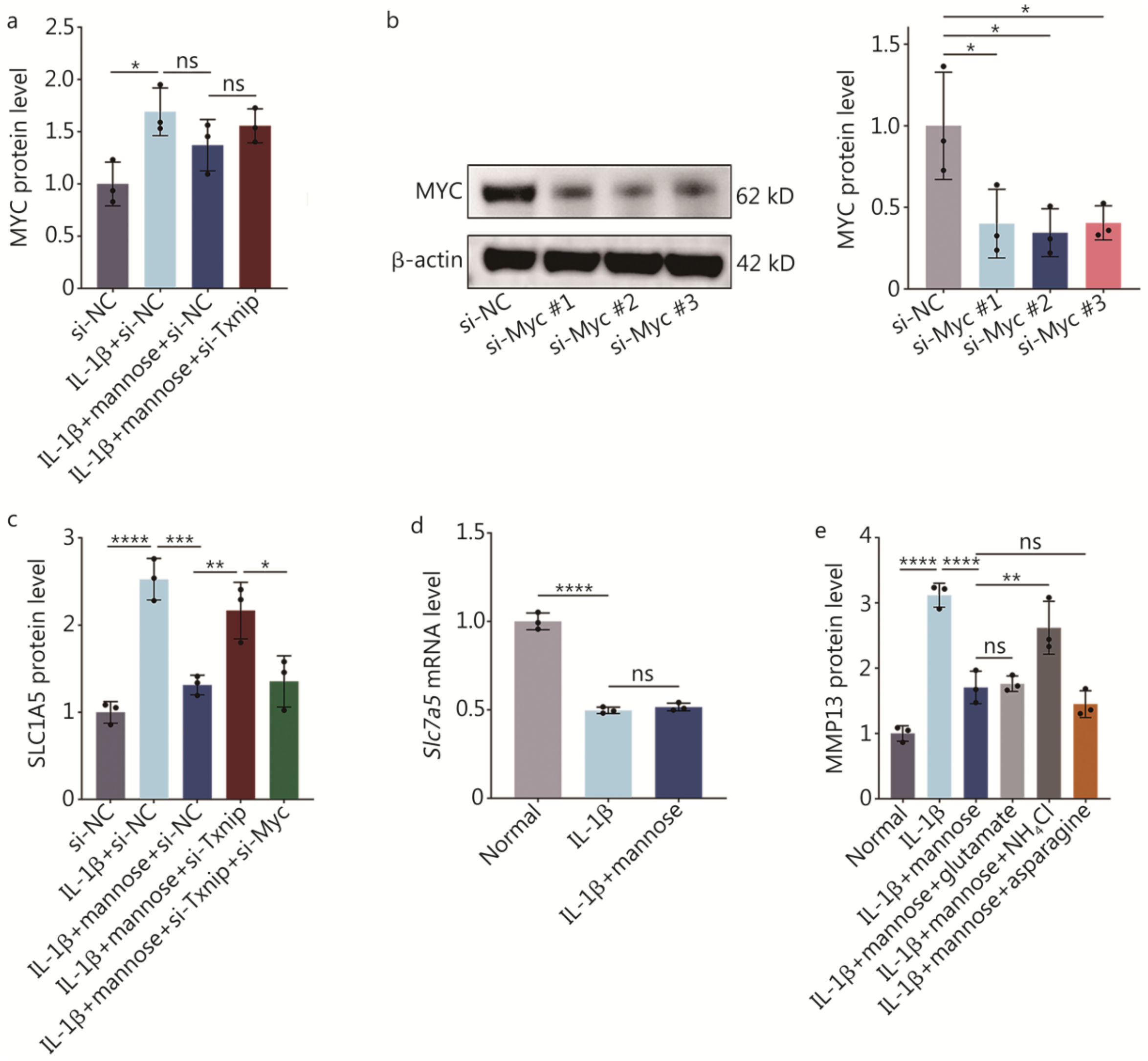

Fig. S8 Western blotting quantitative analysis (**Figs. 5g, j** and **6c**), verification of MYC's small interfering RNA (si-Txnip), and mRNA expression of *Slc7a5*. **a** Western blotting quantitative analysis of MYC in NP cells treated with si-NC, IL-1β + si-NC, IL-1β + mannose + si-NC and IL-1β + mannose + si-Txnip (in **Fig. 5g**). **b** Western blotting and quantitative analysis of MYC in NP cells treated with si-NC and three sequences of different si-Myc. si-Myc #2 was chosen for the following experiments. **c** Western blotting quantitative analysis of SLC1A5 in NP cells treated with si-NC, IL-1β + si-NC, IL-1β + mannose + si-NC, IL-1β + mannose + si-Txnip, IL-1β + mannose + si-Txnip + si-Myc (in **Fig. 5j**). **d** RT-qPCR analysis of *Slc7a5* in NP cells treated with IL-1β and IL-1β + mannose. **e** Western blotting quantitative analysis of MMP13 in NP cells treated with IL-1β, IL-1β + mannose, IL-1β + mannose + glutamate (200 μmol/L)/NH_4Cl (2 mmol/L)/asparagine (1 mmol/L) (in **Fig. 6c**). 10 ng/ml IL-1β and 40 mmol/L mannose were used, $n = 3$. $^{*}P < 0.05$, $^{**}P < 0.01$, $^{***}P < 0.001$, $^{****}P < 0.0001$. ns non-significant, IL-1β interleukin-1β, SLC1A5 solute carrier family 1 member 5, SLC7A5 solute

carrier family 7 member 5, MMP13 matrix metalloproteinase 13, NP nucleus pulposus

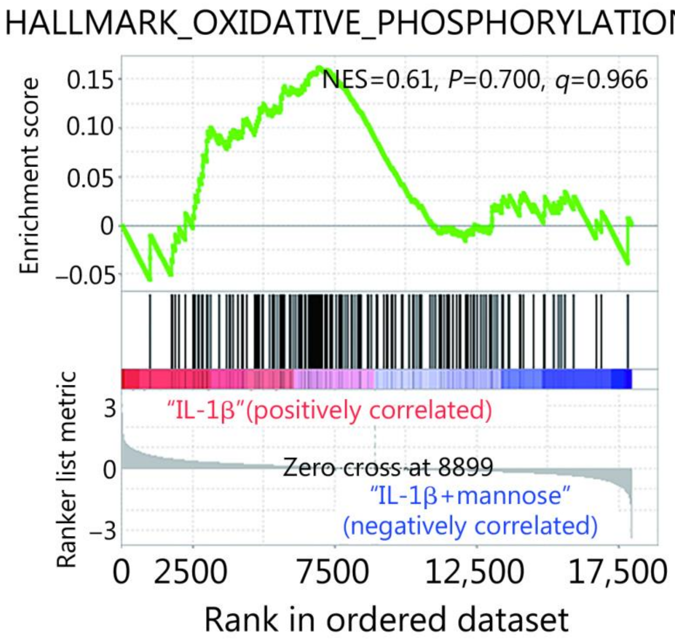

Fig. S9 GSEA analysis of HALLMARK_OXIDATIVE_ PHOSPHORYLATION between the IL-1β group vs. IL-1β + mannose group. GSEA gene set enrichment analysis, IL-1β interleukin-1β, NES normalized enrichment score

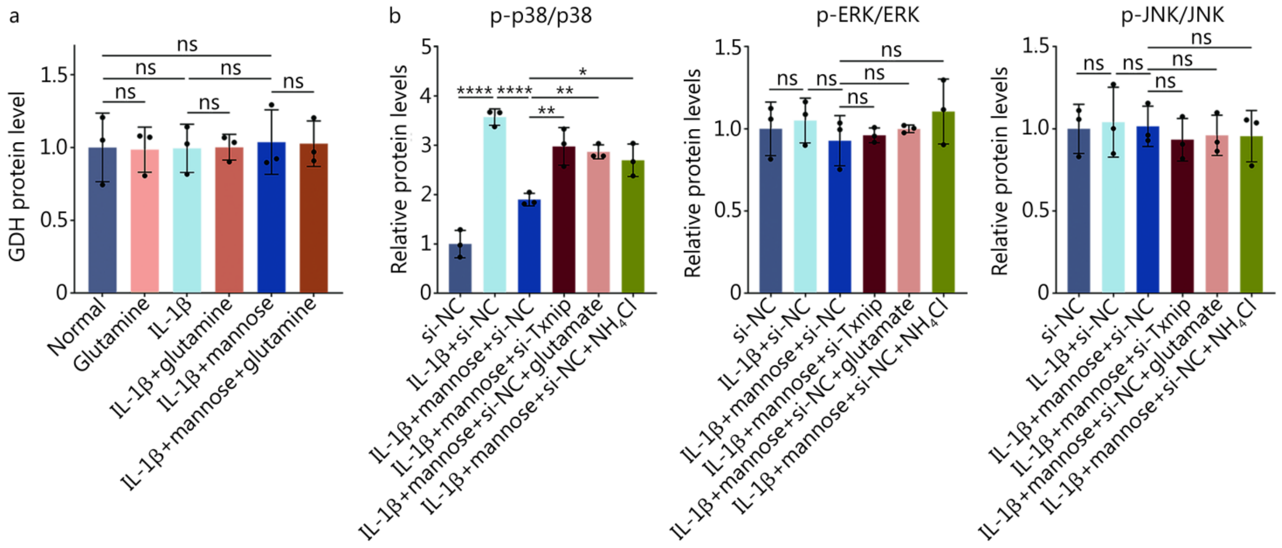

Fig. S10 Western blotting quantitative analysis of **Fig. 6g-h**. **a** Western blotting quantitative analysis of GDH in NP cells treated with glutamine, IL-1β, IL-1β + glutamine, IL-1β + mannose, IL-1β + mannose + glutamine (in **Fig. 6g**). **b** Western blotting quantitative analysis of MAPK pathway key proteins in NP cells treated with si-NC, IL-1β + si-NC, IL-1β + mannose + si-NC, IL-1β + mannose + si-Txnip/si-NC + glutamine/si-NC + NH_4Cl (in **Fig. 6h**). IL-1β 10 ng/ml, mannose 40 mmol/L, and glutamine 8 mmol/L were used, $n = 3$. $^{*}P < 0.05$, $^{**}P < 0.01$, $^{****}P < 0.0001$. ns non-significant, IL-1β interleukin-1β, GDH glutamate dehydrogenase 1, ERK extracellular signal-regulated kinases, JNK c-jun N-terminal kinase, NP nucleus pulposus

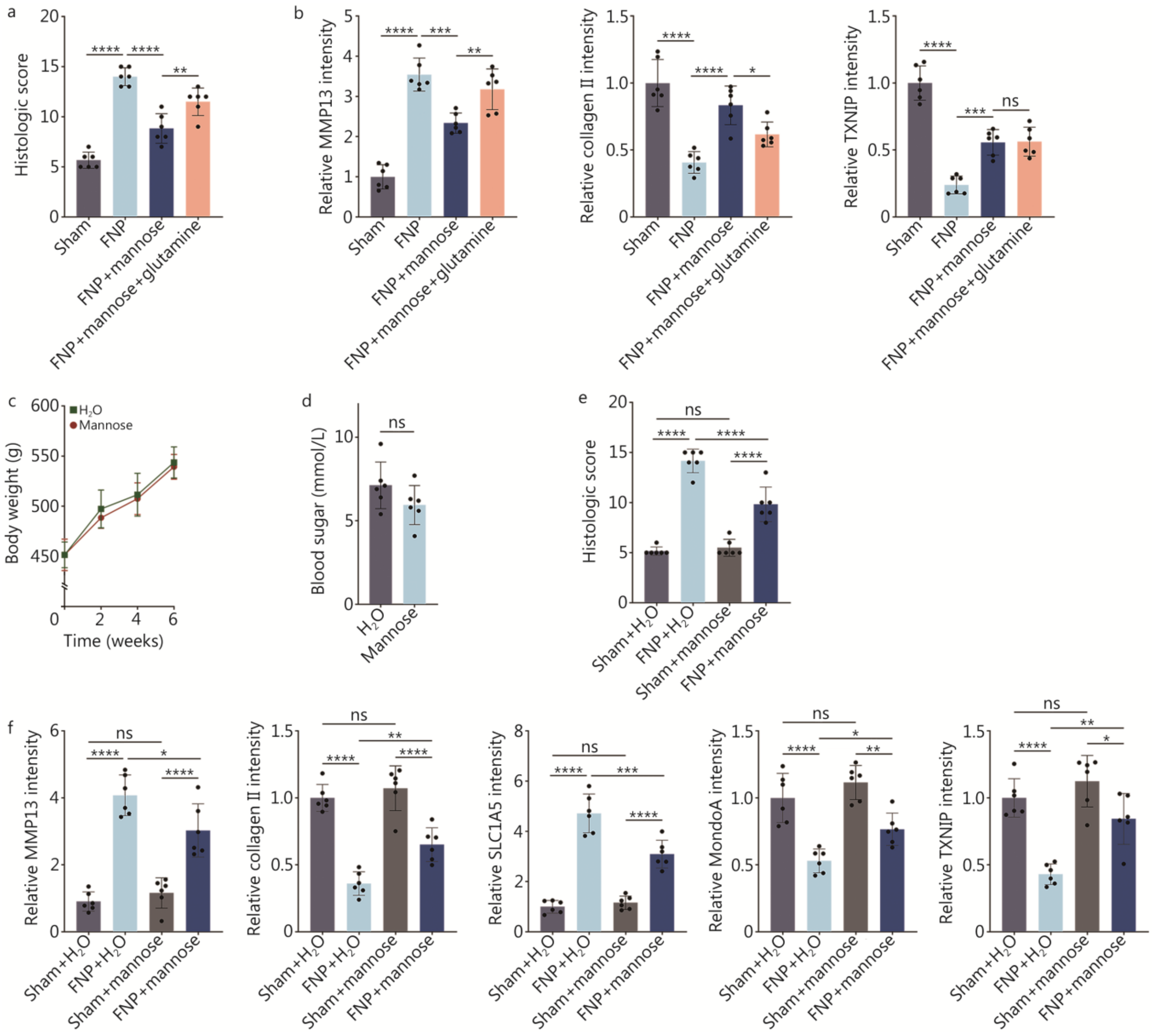

Fig. S11 Quantitative analysis of **Figs. 7-8**, and body weight and blood sugar of water-fed group and mannose-fed group. **a** Histologic score of sham group, FNP group, FNP + mannose group and FNP + mannose + glutamine group. **b** Quantitative analysis of MMP13, collagen II and TXNIP in sham group, FNP group, FNP + mannose group and FNP + mannose + glutamine group. **c** Six-week body weight between water-fed group and mannose-fed group. **d** Blood sugar levels at 6-week endpoint of water-fed group and mannose-fed group. **e** Histologic score of sham + H_2O group, FNP + H_2O group, sham + mannose group, FNP + mannose group. **f** Quantitative analysis of MMP13, collagen II, SLC1A5, MondoA and TXNIP in sham + H_2O group, FNP + H_2O group, sham + mannose group, FNP + mannose group. $n = 6$. $^{*}P < 0.05$, $^{**}P < 0.01$, $^{***}P < 0.001$, $^{****}P < 0.0001$. ns non-significant, FNP fine needle puncture, MMP13 matrix metalloproteinase 13, TXNIP thioredoxin-interacting protein, SLC1A5 solute carrier family 1 member 5, MondoA Max-like protein X interacting protein

Table S1 Primer sequences for RT-qPCR (5’ to 3’)

Gene	Forward	Reverse
Mmp1	GCTTCTTTGGCTTCCCTAGC	CCTGGATCCATGGACTGTGT
Mmp3	TGCTCATGAACTTGGCCACT	GTGGGAGGTCCATAGAGGGAT
Mmp9	CTGTCCAGACCAAGGGTACAG	CAGGTTTAGAGCCACGACCA
Mmp13	CAGATTCTTCTGGCGTCTGC	CTCGGGATGGATGCTCGTAT
Adamts4	TTGAAGAGGTCGGTTCGGTG	CCTGGATCCATGGACTGTGT
Collagen II	AGAGCAAGGAGAAGAAGCACAT	TGGACAGTAGACGGAGGAAAGT
Txnip	AGTGCTCACTCAGAAGCTGT	CTTGGAGCCAGGGACACTAA
Gdh	AGCATCTTGGAGGCTGACT	TGACTCTGGGTGCATTGGAT
Slc1a5	ATCTGGTGTCTGCTTCTGCT	AGAGCCACGCCAAAGACTAR
Slc7a5	ACTGCTACAGTGTGAAGGCT	CTTCTGGTGCAGGTTGGATG
β-actin	CAAGGCTGAGAATGGGAAGC	GAAGACGCCAGTAGACTCCA

Mmp1 matrix metalloproteinase 1, *Mmp3* matrix metalloproteinase 3, *Mmp9* matrix metalloproteinase 9, *Mmp13* matrix metalloproteinase 13, *Adamts4* a disintegrin and metalloproteinase with thrombospondin motifs 4, *Txnip* thioredoxin-interacting protein, *Gdh* glutamate dehydrogenase 1, *Slc1a5* solute carrier family 1 member 5, *Slc7a5* solute carrier family 1 member 5

Table S2 Primer sequences of small interfering RNA (5’ to 3’)

Name	Sense	Antisense
si-NC	UUCUCCGAACGUGUCACGUTT	ACGUGACACGUUCGGAGAATT
si-Txnip #1	GCAAACAGACCUUGGACUATT	UAGUCCAAGGUCUGUUUGCTT
si-Txnip #2	CUGUGAAGGUGAUGACAUUTT	AAUGUCAUCACCUUCACAGTT
si-Txnip #3	CAUGAGACCAGGAAACAAATT	UUUGUUUCCUGGUCUCAUGTT
si-Myc #1	AGAACAAGAUGAUGAGGAATT	UUCCUCAUCAUCUUGUUCUTT
si-Myc #2	GAGGAUAUCUGGAAGAAAUTT	AUUUCUUCCAGAUAUCCUCTT
si-Myc #3	GCGACGAGGAAGAGAAUUUTT	AAAUUCUCUUCCUCGUCGCTT

Table S3 Interaction forces of MondoA and glucose 6-phosphate

Type of interaction	Interaction site	Interaction distance
Hydrogen bond interaction	THR101	3.5 Å、2.1 Å
	LEU120	2.1 Å
	GLN105	2.5 Å
Electrostatic interaction	/	/
Hydrophobic interaction	/	/

Table S4 Interaction forces of MondoA and mannose 6-phosphate

Type of interaction	Interaction site	Interaction distance
Hydrogen bond interaction	LEU120	2.1 Å
	GLN105	2.2 Å、2.3 Å
	THR101	2.3 Å
	SER86	2.0 Å
	ARG89	2.2 Å
Electrostatic interaction	ARG89	2.9 Å、3.0 Å
Hydrophobic interaction	/	/
